# Albiflorin‐Mediated MAP2K1 Targeting and HIF‐1 Signaling Inhibition Contribute to the Therapeutic Efficacy in Hyperuricemia‐Associated Cognitive Impairment

**DOI:** 10.1155/humu/5859468

**Published:** 2026-01-19

**Authors:** Huimin Xiao, Xinwen Huang, Rui Yao, Jincai Liu, Linrui Duan, Siwang Wang, Jinming Gao

**Affiliations:** ^1^ Shaanxi Key Laboratory of Natural Products & Chemical Biology, College of Chemistry & Pharmacy, Northwest A&F University, Xianyang, China, nwsuaf.edu.cn; ^2^ Shaanxi Fengdan Zhengyuan Biotechnology Limited Company, Xi’an, China; ^3^ Department of Life Science and Medicine, Northwest University, Xi’an, China, nwu.edu.cn

**Keywords:** albiflorin, cognitive impairment, HIF-1 signaling pathway, hyperuricemia, MAP2K1, *Paeonia suffruticosa* fruit pod extract

## Abstract

This study investigated the therapeutic effects and mechanisms of *Paeonia suffruticosa* fruit pod extract (EMP) and its main component albiflorin (AF) on hyperuricemia‐associated cognitive impairment (HUA‐CI). A HUA‐CI mouse model was established, with cognitive function evaluated via Morris water maze. Hippocampal pathology, inflammation, oxidative stress, and apoptosis were assessed using HE staining, ELISA, TUNEL, and Western blotting. Network pharmacology predicted EMP’s targets, and molecular docking analyzed AF‐MAP2K1 binding. In vitro experiments used UA‐stimulated BV2 and HT22 cells to explore AF’s effect on HIF‐1 signaling. EMP significantly improved cognitive function and reduced pathological damage in the hippocampus of HUA‐CI mice. It exerted protective effects by inhibiting inflammatory responses, alleviating oxidative stress, and preventing cell apoptosis. Network pharmacology analysis revealed that EMP acts through multiple targets and pathways, particularly via the strong binding affinity between AF and MAP2K1. Both in vivo and in vitro studies demonstrated that AF inhibited the HIF‐1 signaling pathway, thereby reducing microglial activation and associated inflammation, mitigating uric acid‐induced neuronal apoptosis, enhancing antioxidant defenses, and protecting neuronal function. Our research indicates that EMP exerts multi‐target therapeutic effects on HUA‐CI; AF plays a key role by targeting MAP2K1 and inhibiting HIF‐1 signaling.

## 1. Introduction

Hyperuricemia is a metabolic disorder characterized by elevated levels of uric acid (UA) in the bloodstream, exceeding the normal range. Typically, it is defined as serum UA levels exceeding 7 mg/dL (approximately 420 *μ*mol/L) in males and 6 mg/dL (approximately 360 *μ*mol/L) in females [1]. Persistent hyperuricemia can lead to a spectrum of health issues, with the most common being gouty arthritis, an acute inflammatory response caused by the deposition of urate crystals in joints or other tissues. In addition to this, hyperuricemia has been associated with chronic kidney disease, cardiovascular diseases, diabetes, obesity, and other metabolic syndromes [2, 3]. Recent studies have further revealed the potential links between hyperuricemia and neurological disorders, particularly increased risks of cognitive decline and dementia [3].

Cognitive impairment refers to significant functional deterioration in multiple cognitive domains such as memory, learning ability, attention, and language expression [4]. Its etiology is complex and diverse, potentially stemming from neurodegenerative diseases like Alzheimer’s disease or related to non‐neurodegenerative factors such as vascular lesions and metabolic disorders [5]. Notably, several epidemiological surveys and clinical studies have indicated a higher incidence of cognitive impairment in patients with hyperuricemia. For instance, a study by Claudio Tana et al. [6] found a significant correlation between hyperuricemia and poorer cognitive performance. Another study [7] suggested that hyperuricemia is associated with reduced cerebral blood flow caused by heart failure and subsequent cognitive decline. Additionally, research [8] has shown that hyperuricemia is linked to an increased risk of Alzheimer’s disease and vascular dementia. Mechanistically, high levels of UA can cause microvascular damage in the brain, promote oxidative stress and inflammatory responses, thereby impairing neuronal function, disrupting normal neural pathways, and ultimately affecting cognitive performance. Relevant studies [7] have indicated that hyperuricemia is associated with reduced cerebral blood flow and cognitive decline caused by heart failure, and is related to the development of atherosclerotic inflammation and dementia. Furthermore, there is a correlation between hyperuricemia and the risk of Alzheimer’s disease and vascular dementia, but some studies [9] have also found an inverse association between serum UA levels and the risk of Alzheimer’s disease. Some studies [10] have suggested that hyperuricemia may have a protective effect against dementia due to its antioxidant properties; however, hyperuricemia may also promote arteriosclerosis and negatively impact cognitive function. It is evident that there is a complex relationship between hyperuricemia, cognitive dysfunction, dementia, and metabolic syndrome, necessitating further research to elucidate the underlying mechanisms.

In recent years, with the growing interest in natural medicines and increasing concerns about the side effects of synthetic drugs, traditional Chinese medicine (TCM) has gained more application in the treatment of modern diseases due to its unique therapeutic effects and fewer adverse reactions. Numerous studies have shown that plant extracts possess significant anti‐inflammatory and antioxidant activities capable of reducing UA levels. For example, Jiang et al. [11] have revealed the molecular mechanisms of the TCM *Phellodendron amurense* in treating hyperuricemia using network pharmacology and other methods. *Paeonia suffruticosa* Andr, as an important medicinal plant, has been widely used for its root bark in aspects such as blood circulation, pain relief, and anti‐inflammatory effects [12]. The fruit pod of the peony, a part of this plant, also has a long history of folk use, primarily for treating symptoms such as fever‐induced fluid loss and throat pain [13]. Studies have shown that the peony fruit pod is rich in various secondary metabolites, including flavonoids, polyphenols, monoterpene glycosides, and triterpene saponins [14]. These components exhibit multiple bioactivities such as antioxidant, anti‐inflammatory, and antitumor properties [15]. Although there are numerous studies on the chemical composition and pharmacological effects of peony fruit pods, research on their effectiveness in intervening hyperuricemia and improving the resulting cognitive impairment is still lacking. In light of this, the present study employs a combination of network pharmacology and experimental validation to comprehensively elucidate the mechanisms of peony fruit pod in treating hyperuricemia and to assess its impact on cognitive function.

## 2. Materials and Methods

### 2.1. Experimental Animals and Grouping

Male C57BL/6J mice (8 weeks old, weighing 20–25 g) were obtained from Shanghai SLAC Laboratory Animal Co., Ltd. The animals were maintained under controlled conditions of temperature (22°C ± 2°C), humidity (50% ± 10%), and a 12‐h light/dark cycle, with ad libitum access to water and standard chow. The animal experiment was permitted by the Animal Ethics Committee of Northwest University (Approval No. NWUAWC‐20220908M).

Groups were established for drug administration as follows: Control group: Untreated controls. HUA‐CI model group: Hyperuricemia‐cognitive impairment (HUA‐CI) models were induced by intraperitoneal injections of potassium oxonate (200 mg/kg, Sigma‐Aldrich) and hypoxanthine (300 mg/kg, Sigma‐Aldrich) once daily for seven consecutive days. HUA‐CI + EMP groups: Based on the HUA‐CI model, low (12.5 mg/kg/day), medium (50 mg/kg/day), and high (100 mg/kg/day) doses of *Paeonia suffruticosa* fruit pod extract (EMP, provided by Chengdu Precision Biotech Co. Ltd., batch number: PRF‐202312) were administered intraperitoneally for 7 days. EMP doses (12.5, 50, 100 mg/kg/day) were selected based on acute toxicity tests (no mortality observed up to 500 mg/kg) and pilot experiments showing dose‐dependent reductions in serum UA levels (Figure 1a). HUA‐CI + AF group: albiflorin (AF, Sigma‐Aldrich, catalog number: A14007) was administered at a dose of 100 mg/kg/day to the HUA‐CI model mice. HUA‐CI + AF + FG4592 group: AF (100 mg/kg/day) and the HIF‐1*α* activator FG4592 (10 mg/kg/day, Selleck Chemicals, catalog number: S7665) were given to the HUA‐CI model mice. HUA‐CI + YC‐1 group: The HIF‐1*α* inhibitor YC‐1 (5 mg/kg/day, MedChemExpress, catalog number: HY‐10064) was administered to the HUA‐CI model mice. All drugs were dissolved in either physiological saline or DMSO and administered intraperitoneally for 7 days.

Figure 1Effects of EMP on serum uric acid levels, renal function, and uric acid transporters in HUA‐CI mice (a). Serum uric acid levels in mice treated with vehicle (Control), HUA‐CI, or EMP (12.5, 25, 50, 100, 200 mg/kg/day). (b) Xanthine oxidase (XOD) activity in serum. (c) Renal function markers (creatinine [CRE] and blood urea nitrogen [BUN]) in serum. (d) Western blot analysis of renal uric acid transporters (GLUT9, URAT1, ABCG2, OAT1) normalized to GAPDH. *n* = 5. ∗*p* < 0.05, ∗∗*p* < 0.01, ∗∗∗*p* < 0.001.

**Figure:**
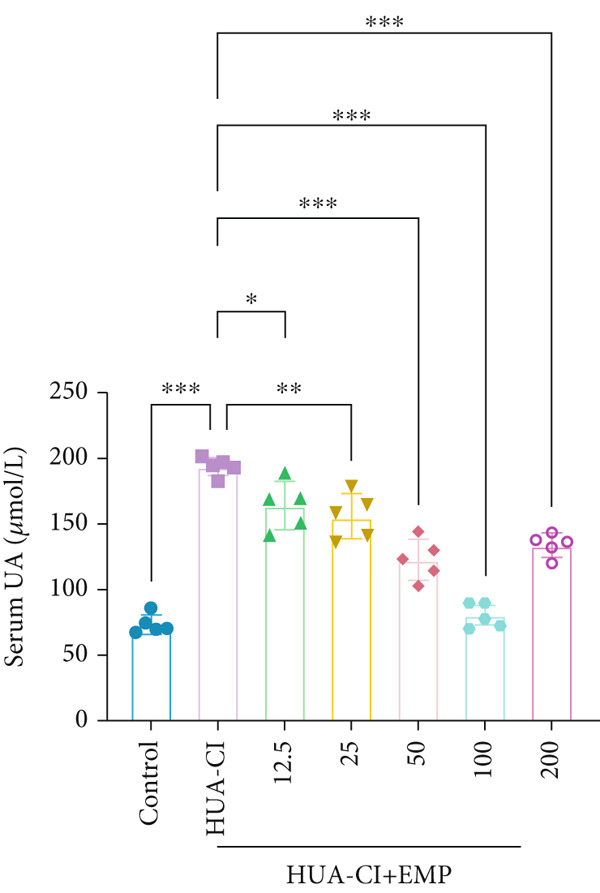
(a)

**Figure:**
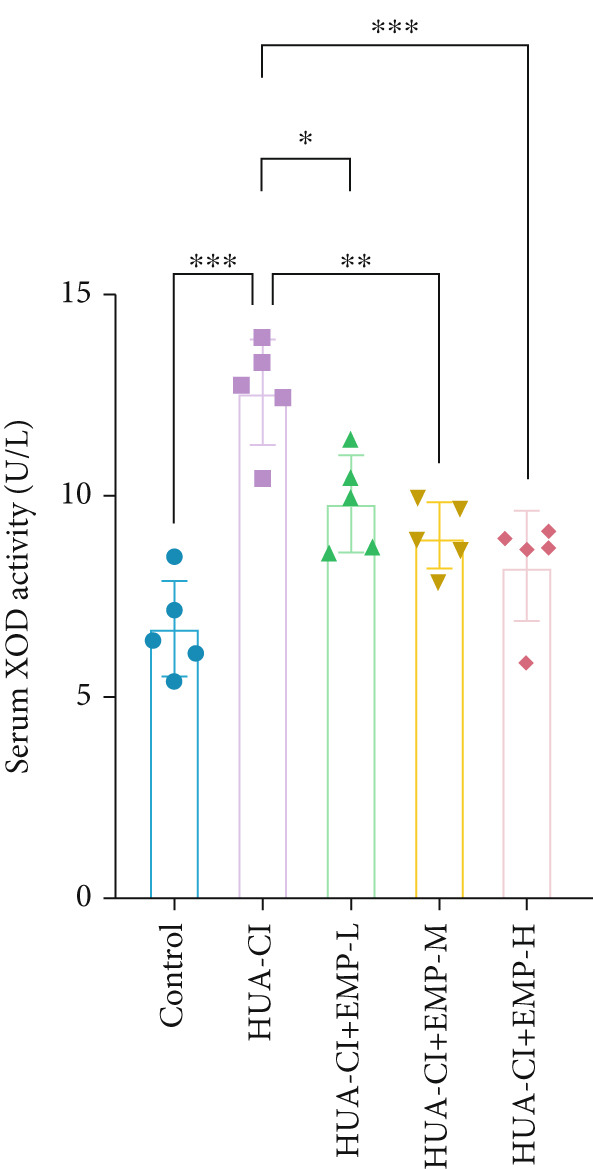
(b)

**Figure:**
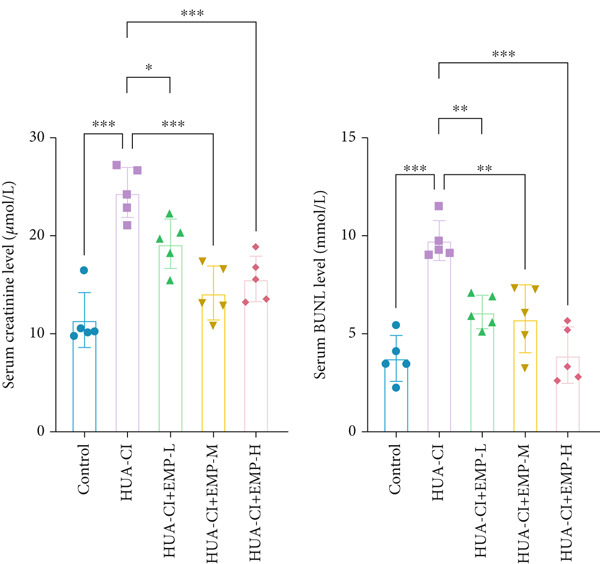
(c)

**Figure:**
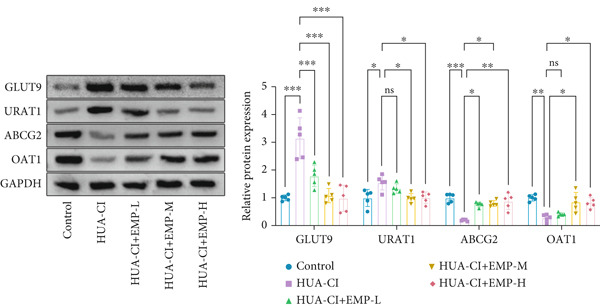
(d)

### 2.2. Detection of Uric Acid, Creatinine, and Blood Urea Nitrogen in Serum

To separate the supernatant from the precipitate, blood samples were centrifuged at 3500 rpm for 15 min at 4°C. The UA concentration was measured using an enzymatic colorimetric method following the kit instructions (Shanghai Rongsheng Biopharmaceutical Co. Ltd., Shanghai, China). For creatinine detection, a creatinine assay kit was used with a sarcosine oxidase method (Wuhan Elabscience Biotechnology Co. Ltd., Wuhan, China). Blood urea nitrogen levels were determined using a urea nitrogen assay kit with a urease method, following the kit instructions (Wuhan Elabscience Biotechnology Co. Ltd., Wuhan, China).

### 2.3. Detection of Serum and Hepatic Xanthine Oxidase (XOD) Activity

The activity of XOD in serum was determined using an XOD test kit in accordance with the manufacturer’s instructions (Nanjing Jiancheng Bioengineering Institute, Nanjing, China).

### 2.4. Behavioral Testing

Morris Water Maze Test: A circular water maze (120 cm in diameter, 30 cm deep) with a hidden platform submerged 1.5 cm below the surface was employed. Mice underwent four training trials per day for five consecutive days, each lasting 60 s. Escape latency and swim paths were recorded using the EthoVision XT video tracking system.

### 2.5. Tissue Collection and Processing

Following behavioral testing, mice were anesthetized with sodium pentobarbital and perfused transcardially with 4% paraformaldehyde. Brains were extracted, and the hippocampal region was isolated for further analysis. Detailed procedures included rapid thoracotomy postanesthesia, followed by flushing blood with cold PBS using a 20 mL syringe connected to a cannula inserted into the left ventricle, then perfusion with 4% paraformaldehyde. After 10 min, brains were removed and fixed in 4% paraformaldehyde for 4 h before being transferred to 30% sucrose solution overnight for dehydration. Sections of 20 *μ*m thickness were prepared using a cryostat (Leica CM1860) and stored in antifreeze solution until use.

### 2.6. Network Pharmacology Studies

#### 2.6.1. Prediction of Targets for Active Components

Isomeric SMILES codes for major active components in EMP were retrieved from PubChem, and potential targets were predicted using Swiss Target Prediction. Targets were screened with the criterion of “probability > 0.05” to ensure potential relevance. Among the 21 identified EMP components, one component had no predicted targets, and the remaining 20 components collectively yielded 230 potential targets after removing duplicates. A network linking drugs, active components, and their targets was constructed following screening.

#### 2.6.2. Collection of Disease‐Associated Targets

Target information related to hyperuricemia was collected from Genecards, while target information associated with cognitive impairment was gathered from OMIM. Additional target information was sourced from Drugbank. Duplicate entries were removed to identify common targets.

#### 2.6.3. PPI Network Construction

Common key targets were identified by comparing disease targets with those of EMP’s active components. A protein–protein interaction (PPI) network model was built using String (confidence score ≥ 0.4) and Cytoscape, with core targets selected based on higher degree values.

#### 2.6.4. Functional and Pathway Enrichment Analysis

GO (Gene Ontology) and KEGG (Kyoto Encyclopedia of Genes and Genomes) pathway enrichment analyses were performed on the 24 common key targets to uncover biological processes and signaling pathways. GO analysis was conducted using DAVID Bioinformatics Resources 6.8, with significance defined by adjusted *p* < 0.05 (Benjamini–Hochberg correction) and gene count ≥ 5. KEGG pathway analysis was executed using the same criteria (adjusted *p* < 0.05, gene count ≥ 5) and visualized via bubble charts (GO) and pathway maps (KEGG). To ensure consistency, both analyses employed uniform color schemes (green‐red gradients) and removed ambiguous “has” annotations.

#### 2.6.5. Component–Target–Pathway Network Diagram Construction

To illustrate the complex interactions between EMP’s active components, their targets, and signaling pathways, a “component–target–pathway” relationship network diagram was constructed using Cytoscape. Nodes representing active components, targets, and pathways were defined, and edge color attributes were set according to interaction types (e.g., activation or inhibition). Node sizes were adjusted based on degree values to highlight core targets within the network.

#### 2.6.6. Molecular Docking Simulation

Molecular docking simulations were carried out using AutoDock Vina to investigate the binding mode and affinity between AF and MAP2K1 (MEK). The 3D structure of AF was retrieved from PubChem and optimized, while the crystal structure of MAP2K1 was obtained from the PDB database and processed to remove nonessential elements. In AutoDock Vina, a spatial region encompassing the potential binding pocket was defined, and appropriate grid spacing was set to ensure computational accuracy. Following docking initiation, multiple AF‐MAP2K1 complex models were generated, and the binding free energy of each conformation was calculated. The conformation with the highest score was selected as the optimal solution. Visualization of hydrogen bonds and hydrophobic interactions between AF and critical amino acid residues of MAP2K1 was performed using Discovery Studio Visualizer.

### 2.7. Culture and Treatment of Microglia

BV2 microglial cells (Type Culture Collection of the Chinese Academy of Sciences, Shanghai, China) were cultured in DMEM (Thermo Fisher Hyclone, Utah, United States) supplemented with 10% FBS (Thermo Fisher Scientific, MA, United States) and 1% penicillin‐streptomycin at 37°C in 5% CO₂. Cells were seeded at 5 × 10^5^ cells/well in 6‐well plates and treated as follows: Control (no UA, 0.1% DMSO vehicle), HUA‐CI (1 mM UA for 24 h), HUA‐CI + AF (1 mM UA + 10 *μ*M AF), HUA‐CI + YC‐1 (1 mM UA+5 *μ*M YC‐1), and HUA‐CI + AF + FG4592 (1 mM UA+100 *μ*M AF+10 *μ*M FG4592). All drugs were dissolved in DMSO (final concentration ≤ 0.1%) and added 1 h prior to UA exposure. After 24 h, cells were harvested for RNA/protein extraction or functional assays.

### 2.8. CCK‐8 Assay for BV2 Cells Viability

Cell viability was assessed using the Cell Counting Kit‐8 (CCK‐8, Dojindo Laboratories, catalog number: CK04). BV2 cells were seeded into 96‐well plates at a density of 1 × 10^4^ cells per well and incubated for 24 h. Fresh medium containing different treatment drugs was then added, and the cells were further incubated for an additional 24 h. Subsequently, 10 *μ*L of CCK‐8 solution was added to each well, followed by incubation at 37°C for 1 h. Absorbance was measured at 450 nm using a microplate reader (Thermo Scientific Multiskan FC).

### 2.9. MTT Assay for HT22 Neuronal Cell Viability Treated With BV2 CM

HT22 hippocampal neuronal cells (Type Culture Collection of the Chinese Academy of Sciences, Shanghai, China) were cultured in DMEM supplemented with 10% FBS and 1% penicillin‐streptomycin at 37°C in 5% CO₂. Conditioned medium (CM) from LPS‐activated BV2 microglia (1 *μ*g/mL LPS for 24 h) was collected, centrifuged to remove debris, and used to treat HT22 cells in 96‐well plates (1 × 10^4^ cells/well). Groups included: Control (no CM exposure), HUA‐CI (100% BV2 CM), HUA‐CI + AF (100% BV2 CM + 100 *μ*M AF), and HUA‐CI + AF + FG4592 (100% BV2 CM+100 *μ*M AF +10 *μ*M FG4592). After 24 h treatment, 20 *μ*L MTT (5 mg/mL) was added, followed by 4 h incubation. DMSO (150 *μ*L) solubilized formazan crystals, and absorbance was measured at 490 nm. Cell viability was calculated as: (treated absorbance/control absorbance) × 100*%*.

### 2.10. PCR and Western Blotting Analysis

#### 2.10.1. RNA Extraction and Real‐Time Quantitative PCR

Total RNA was extracted from tissues or cells using TRIzol reagent (Invitrogen, catalog number: 15596026). After homogenization in 1 mL of TRIzol, samples were left at room temperature for 5 min. Chloroform (200 *μ*L per 1 mL of TRIzol) was added, and the mixture was vigorously shaken for 15 s before being left at room temperature for 3 min. Samples were centrifuged at 12,000 g for 15 min, and the aqueous phase was transferred to a new tube. Isopropanol (500 *μ*L per 1 mL of TRIzol) was added, mixed by inversion, and left at room temperature for 10 min. Following a 10‐min centrifugation at 12,000 g, the supernatant was discarded, and the RNA pellet was washed twice with 75% ethanol. The RNA was air‐dried and dissolved in RNase‐free water. RNA concentration was determined, and cDNA was synthesized. Real‐time quantitative PCR was performed using SYBR Green PCR Master Mix (Applied Biosystems, catalog number: 4309155). Primer sequences: 5 ^′^‐GAAGCGAATGCTGGAGAAA‐3 ^′^ (forward) and 5 ^′^‐GACCAGTTGGCCTCTTGTGT‐3 ^′^ (reverse) for IBA1; Primer sequences: 5 ^′^‐CTGATCTCAGATGCTGTTTCC‐3 ^′^ (forward) and 5 ^′^‐AGCCTTTGTAAATGGGCAC‐3 ^′^ (reverse) for CD86; Primer sequences: 5 ^′^‐GAACTGAAAGGAAAGTTCCCA‐3 ^′^ (forward) and 5 ^′^‐AATGTACACGATGTCTTTGGC‐3 ^′^ (reverse) for ARG1; Primer sequences: 5 ^′^‐ACTCTTCCACCTTCGATGC‐3 ^′^ (forward) and 5 ^′^‐CCGTATTCATTGTCATACCAGG‐3 ^′^ (reverse) for GAPDH.

#### 2.10.2. Protein Extraction and Western Blotting

Total protein was extracted from tissues or cells using RIPA lysis buffer (Beyotime, catalog number: P0013B). An appropriate amount of RIPA buffer was added to the lysed samples, which were thoroughly mixed. Protease inhibitor cocktail (Roche, catalog number: 11836153001) was added, and samples were lysed on ice for 30 min. After centrifugation at 12,000 g for 15 min, the supernatant was collected. Protein concentration was quantified using a BCA Protein Assay Kit (Thermo Fisher Scientific, catalog number: 23225). Equal amounts of protein samples were subjected to SDS‐PAGE and transferred to PVDF membranes (Millipore, catalog number: IPVH00010), which were blocked with 5% nonfat milk for 1 h. Membranes were incubated overnight at 4°C with primary antibodies against GLUT9 (Abcam, catalog number: ab223470, 1:500 dilution), U2AF1 (Abcam, catalog number: ab172614, 1:1000 dilution), ABCG2 (Abcam, catalog number: ab207732, 1:1000 dilution), OAT1 (Abcam, catalog number: ab172614, 1:500 dilution), HIF‐1*α* (Abcam, catalog number: ab1, 1:10000 dilution), Bax (Abcam, catalog number: ab32503, 1:1000 dilution), and Caspase‐3 (Abcam, catalog number: ab32351, 1:5000 dilution). IBA1 (Abcam, catalog number: ab178846, 1:10000 dilution), CD86 (Abcam, catalog number: ab239075, 1:1000 dilution), ARG1 (Abcam, catalog number: ab133543, 1:20000 dilution), MAP2K1 (Abcam, catalog number: ab32134, 1:50000 dilution), p‐ERK (Abcam, catalog number: ab201015, 1:1000 dilution), ERK (Abcam, catalog number: ab32537, 1:1000 dilution), and GAPDH (Abcam, catalog number: ab8245, 1:5000 dilution). On the following day, horseradish peroxidase (HRP)‐conjugated secondary antibodies were added, and chemiluminescent detection was performed using ECL.

### 2.11. ELISA Analysis

Inflammatory cytokines IL‐1*β*, IL‐6, TNF‐*α* (RayBiotech, catalog numbers: ELM‐IL1B‐1, ELM‐IL6‐1, ELM‐TNF‐1), and oxidative stress‐related enzymes SOD, CAT, and MDA (Beyotime, catalog numbers: S0139, S0055, S0138) were measured using ELISA kits according to the manufacturer’s instructions. Standards and samples were added to 96‐well plates at 100 *μ*L per well. Biotinylated antibodies were added, and the plate was incubated at 37°C for 1 h. After five washes, HRP‐conjugated streptavidin was added and incubated at 37°C for 30 min. Following another five washes, substrate TMB was added and incubated at 37°C in the dark for 15 min. Stop solution was then added, and absorbance was measured at 450 nm using a microplate reader (Thermo Scientific Multiskan FC). Concentrations of target proteins or factors in the samples were calculated based on standard curves.

### 2.12. Apoptosis Detection

#### 2.12.1. TUNEL Staining

Cell apoptosis was detected using the In Situ Cell Death Detection Kit (Roche, catalog number: 11684795910). Sections were deparaffinized to water and washed three times with PBS for 5 min each. They were treated with proteinase K (20 *μ*g/mL) and washed again with PBS. TUNEL reaction mixture was prepared according to the kit instructions, applied to sections, and incubated at 37°C in the dark for 1 h. Sections were washed three times with PBS and counterstained with DAPI (Vector Laboratories, catalog number: H‐1200). Slides were mounted and examined under a fluorescence microscope.

#### 2.12.2. Flow Cytometry

Apoptosis analysis was conducted using the Annexin V‐FITC/PI Apoptosis Detection Kit (BD Biosciences, catalog number: 556547). HT22 neuronal cells in logarithmic growth phase were harvested by centrifugation and washed twice with precooled PBS. Cells were resuspended in 1× binding buffer at a density of 1 × 10^6^ cells/mL. A 100‐*μ*L aliquot of cell suspension was taken, and 5 *μ*L of Annexin V‐FITC and 5 *μ*L of PI were added. The mixture was gently vortexed and incubated at room temperature in the dark for 15 min. After adding 400 *μ*L of 1× Binding Buffer, flow cytometric analysis was immediately performed using a BD FACSCalibur instrument, and data were analyzed using FlowJo software.

### 2.13. Cellular Thermal Shift Assay

The binding of AF to the MAP2K1 protein was assessed using the CETSA method. Total BV2 cell lysates were equally divided into AF and DMSO groups, incubated at 37°C for 1.5 h, and then aliquoted into 7 PCR tubes. These tubes were heated to 40°C, 50°C, 60°C, 70°C, 80°C, 90°C, and 100°C (a temperature range covering the typical thermal denaturation interval of proteins) for 5 min each. The temperature points were selected based on preliminary pre‐experiments showing that MAP2K1 undergoes gradual thermal denaturation between 40°C and 100°C, which allows clear differentiation of protein stability changes induced by ligand binding. These tubes were heated to seven different temperatures for 5 min each. After centrifugation at 12,000 rpm for 15 min, the supernatant was collected for Western blotting analysis.

### 2.14. Statistical Analysis

Data are expressed as mean ± SEM and were analyzed using GraphPad Prism 8 software. One‐way ANOVA followed by Tukey’s post hoc test was used for intergroup comparisons. For non‐normally distributed data, Mann–Whitney *U* or Kruskal–Wallis tests were applied. Differences were considered statistically significant at *p* < 0.05.

## 3. Results

### 3.1. Pomegranate Peel Extract Alleviates Symptoms in HUA‐CI Mice

To determine the optimal dosage of EMP in mice, we performed a dose‐response experiment in HUA‐CI mice and measured serum UA levels. As shown in Figure 1a, the HUA‐CI model group exhibited significantly higher UA levels than the control group (*p* < 0.01). In the treatment groups, UA levels decreased slightly at 12.5 mg/kg/day (*p* < 0.05), more markedly at 50 mg/kg/day (*p* < 0.01), and most significantly at 100 mg/kg/day (*p* < 0.001). Unexpectedly, at 200 mg/kg/day, UA levels also decreased significantly (*p* < 0.01), which might be attributed to target pathway saturation or pharmacokinetic alterations. Consequently, we designated 12.5, 50, and 100 mg/kg/day as the low, medium, and high doses, respectively. Figure 1b illustrates the impact of different EMP doses on serum XOD activity. Compared with the control group, the HUA‐CI model group displayed significantly elevated XOD activity (*p* < 0.01). EMP treatment resulted in a dose‐dependent reduction in XOD activity. Given that creatinine (CRE) and blood urea nitrogen (BUN) are pivotal biomarkers of renal function, we measured serum CRE and BUN levels to assess the effects of EMP on renal function in mice. Compared with the control group, the HUA‐CI group showed markedly elevated serum CRE and BUN levels. EMP treatment led to a significant, dose‐dependent reduction in these levels (*p* < 0.01), indicating that EMP mitigated renal dysfunction in HUA‐CI mice (Figure 1c). Excessive UA production is frequently attributable to diminished UA excretion, which arises from abnormal renal UA transporters. Hence, we employed Western blot to examine the protein expression of GLUT9, URAT1, ABCG2, and OAT1 in renal tissues. Compared with the control group, the HUA‐CI model group demonstrated increased protein levels of GLUT9 and URAT1, whereas EMP treatment downregulated their expression. Conversely, the protein levels of ABCG2 and OAT1 were reduced in the HUA‐CI model group while EMP treatment dose‐dependently increased their expression (Figure 1d). The above findings confirm the successful establishment of the hyperuricemic mouse model and demonstrate that EMP can reduce UA levels, improve renal function, and alleviate hyperuricemia symptoms.

To investigate the effects of EMP on hyperuricemia‐associated cognitive impairment (HUA‐CI) mice, a series of experiments were conducted to evaluate its impact on cognitive function, reduction of hippocampal pathological damage, suppression of inflammatory responses, decrease in oxidative stress, and prevention of apoptosis. We monitored changes in body weight among groups (Figure 2a). The results indicated no significant differences in body weight across all groups. In the Morris water maze test, untreated HUA‐CI mice exhibited reduced activity levels and impaired spatial learning and memory. Conversely, EMP‐treated mice showed significant improvements in these behavioral deficits, particularly in the high‐dose EMP group, where performance approached that of the Control group (Figure 2b). Further examination via HE staining revealed marked neuronal loss and structural disorganization in the hippocampus of HUA‐CI mice (Figure 2c); however, EMP treatment significantly mitigated these pathological changes, especially at higher doses, suggesting a protective effect of EMP on hippocampal neurons. ELISA analysis was performed to assess inflammatory cytokine levels (Figure 2d) and oxidative stress indicators (Figure 2e) in the hippocampus. Elevated levels of IL‐1*β*, IL‐6, and TNF‐*α* were observed in HUA‐CI mice, which were effectively reduced by EMP treatment. Additionally, increased SOD and CAT activities along with decreased MDA levels indicated that EMP enhances antioxidant defense mechanisms and reduces oxidative stress. In addition, apoptosis experiment (Figure 2f) found that the addition of EMP could reduce apoptosis, especially in the high‐dose group. Western Blotting experiment (Figure 2g) found that the expressions of pro‐apoptotic proteins Bax and Caspase‐3 were significantly decreased after EMP was added, and the effect was most obvious in the high‐dose group. It was further proved that EMP could inhibit apoptosis. Collectively, these findings suggest that EMP exerts multifaceted benefits in HUA‐CI mice, including improved cognitive function, reduced hippocampal pathology, suppressed inflammation, decreased oxidative stress, and prevention of apoptosis.

Figure 2Effects of EMP on Symptoms in HUA‐CI Mice (a). Changes in body weight of mice across all groups throughout the experiment. (b) Morris water maze test evaluating spatial learning and memory capabilities in mice from each group. (c) Hematoxylin and eosin (HE) staining examining pathological changes in the hippocampal region. Scale bar: 50 *μ*m. (d) ELISA analysis measuring levels of inflammatory cytokines in hippocampal tissue. (e) ELISA analysis measuring oxidative stress markers in hippocampal tissue. (f) Flow cytometry assessing apoptosis in the hippocampal region. (g) Western blotting detecting expression of apoptosis‐related proteins in hippocampal tissue. *n* = 5. ∗*p* < 0.05, ∗∗*p* < 0.01, ∗∗∗*p* < 0.001.

**Figure:**
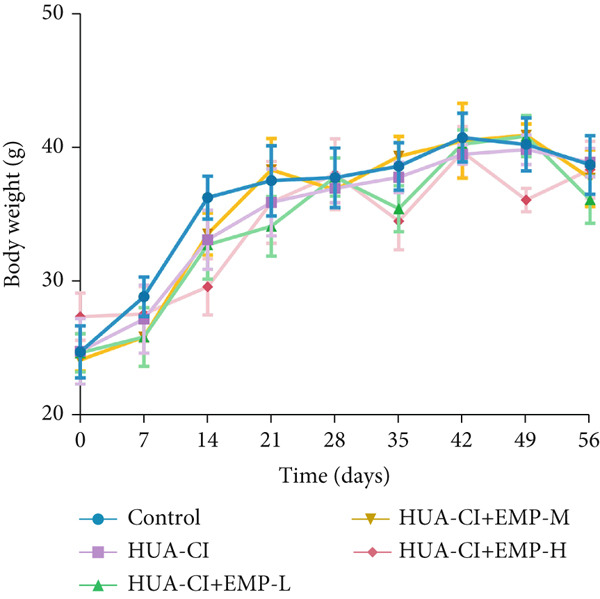
(a)

**Figure:**
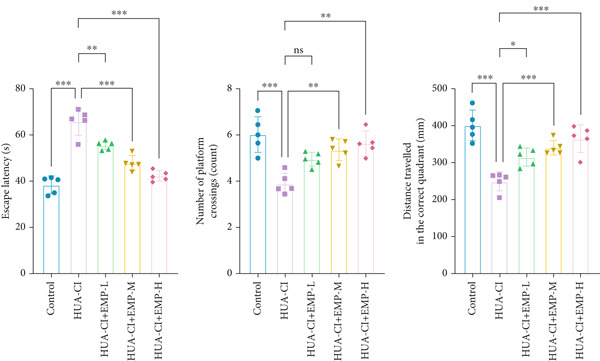
(b)

**Figure:**
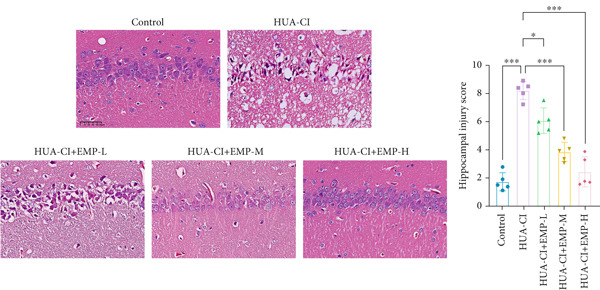
(c)

**Figure:**
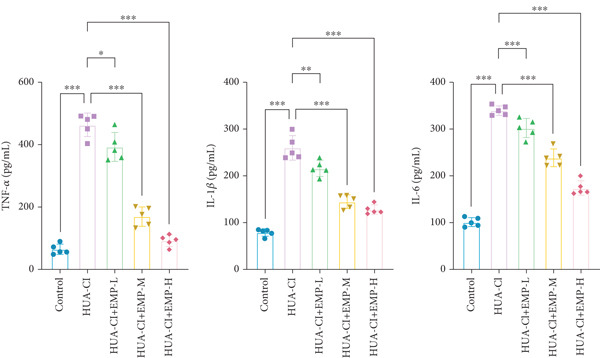
(d)

**Figure:**
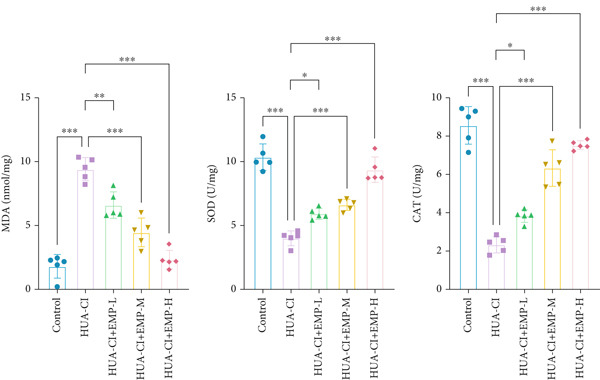
(e)

**Figure:**
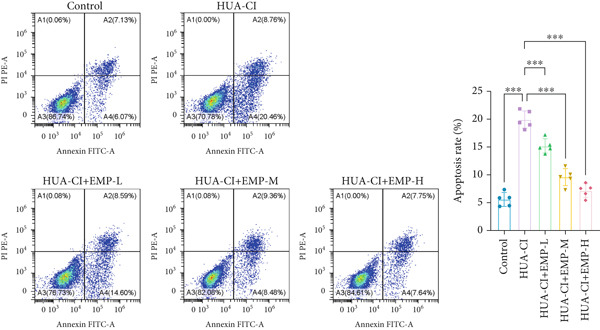
(f)

**Figure:**
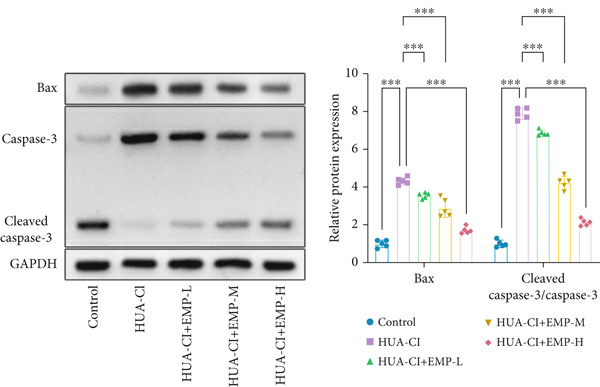
(g)

### 3.2. Network Pharmacology Analysis of EMP′s Potential Mechanisms in Treating HUA‐CI

A total of 21 components in EMP were identified through techniques like HPLC‐MS (detailed in Supporting Table S1). To explore the potential mechanisms underlying EMP’s therapeutic effects on HUA‐CI, we first predicted the targets of EMP’s major active components (Supporting Figure S1). Isomeric SMILES codes for each component were obtained from PubChem, and their potential targets were predicted using Swiss Target Prediction. As per the screening criterion (probability > 0.05) using Swiss Target Prediction, 230 potential targets were identified from 20 EMP components (one component had no predicted targets), with AF and other 19 components all meeting the probability threshold (Table 1). A network linking drugs, active components, and target proteins was constructed (Supporting Figure S1). Subsequently, target information related to hyperuricemia and cognitive impairment was collected from Genecards, OMIM, and Drugbank databases, yielding 1434 and 1506 unique targets, respectively. Intersection of these targets resulted in 347 common targets (Figure 3a), providing critical insights for further research. Comparison of disease targets with EMP’s active component targets identified 24 shared key targets (Figure 3b). The PPI network model was constructed using String and Cytoscape (Figure 3c). The top 10 key targets ranked by degree are shown in Table 2. These targets are likely crucial in treating HUA‐CI. GO enrichment functional analysis (Figure 3d) and KEGG pathway enrichment analysis (Figure 3e) of the 24 common targets revealed involvement in multiple biological processes, including inflammatory response, apoptosis regulation, and oxidative stress response, with enrichment in pathways like HIF‐1 signaling pathway, MAPK signaling pathway, and PI3K‐Akt signaling pathway. Based on this analysis, a “component–target–pathway” relationship network diagram was constructed using Cytoscape (Figure 3f, Supporting Figure S2), visually illustrating the connections between EMP’s active components and their targets. Notably, AF, one of the main active components, interacts with key targets such as HSPA8, VEGFA, SERPINE1, and MAP2K1. Molecular docking simulations of AF with MAP2K1 (Figure 3g) demonstrated a binding energy of −7.472 kcal/mol, indicating strong binding affinity. Overall, network pharmacology analysis suggests that EMP may exert its therapeutic effects on HUA‐CI through multi‐target and multi‐pathway mechanisms, particularly by modulating key targets like MAP2K1, with AF being a primary active component.

**Table 1:** Basic information of main compounds screened by EMP.

**Abbreviation**	**Ingredient**	**No. of targets**
AF	Albiflorin	74
AN	Aesculetin	61
GL	Gallogen	57
CA	Caffeic acid	49
PB	Proanthocyanidins B2	47
MG	Methyl gallate	31
PA	p‐Coumaric acid	29
PL	Paeonol	27
GA	Gallic acid	25
MC	Mudanpioside C	25
PN	Paeoniflorin	24
NP	Apigenin 7‐O‐neohesperidoside	23
AG	Apigenin‐7‐O‐glucoside	22
PG	1,2,3,4,6‐O‐pentagalloyl glucose	20
FA	Ferulic acid	19
HP	Hydroxypaeoniflorin	17
BA	Benzoic acid	13
PC	Pyrocatechol	11
GG	1,2,3,6‐tetra‐O‐galloyl‐*β*‐D‐glucose	8
DP	2,4‐dihydroxyacetophenone	4
VB	Vitamin B6	0

Figure 3Potential mechanisms of EMP in treating HUA‐CI based on network pharmacology (a). Disease target screening. (b) Venn diagram comparing drug and disease targets. (c) Protein–protein interaction (PPI) network of 24 key common targets. (d) Gene Ontology (GO) enrichment functional analysis. (e) Kyoto Encyclopedia of Genes and Genomes (KEGG) pathway enrichment analysis. (f) Drug‐target‐pathway network. (g) Molecular docking simulation of AF with MAP2K1.

**Figure:**
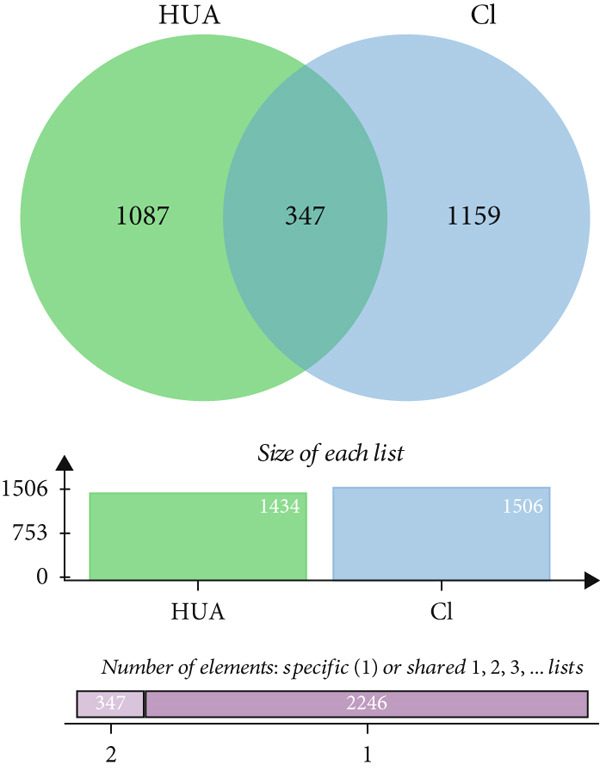
(a)

**Figure:**
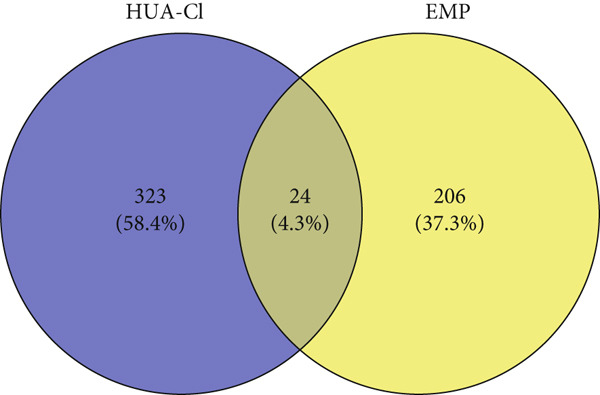
(b)

**Figure:**
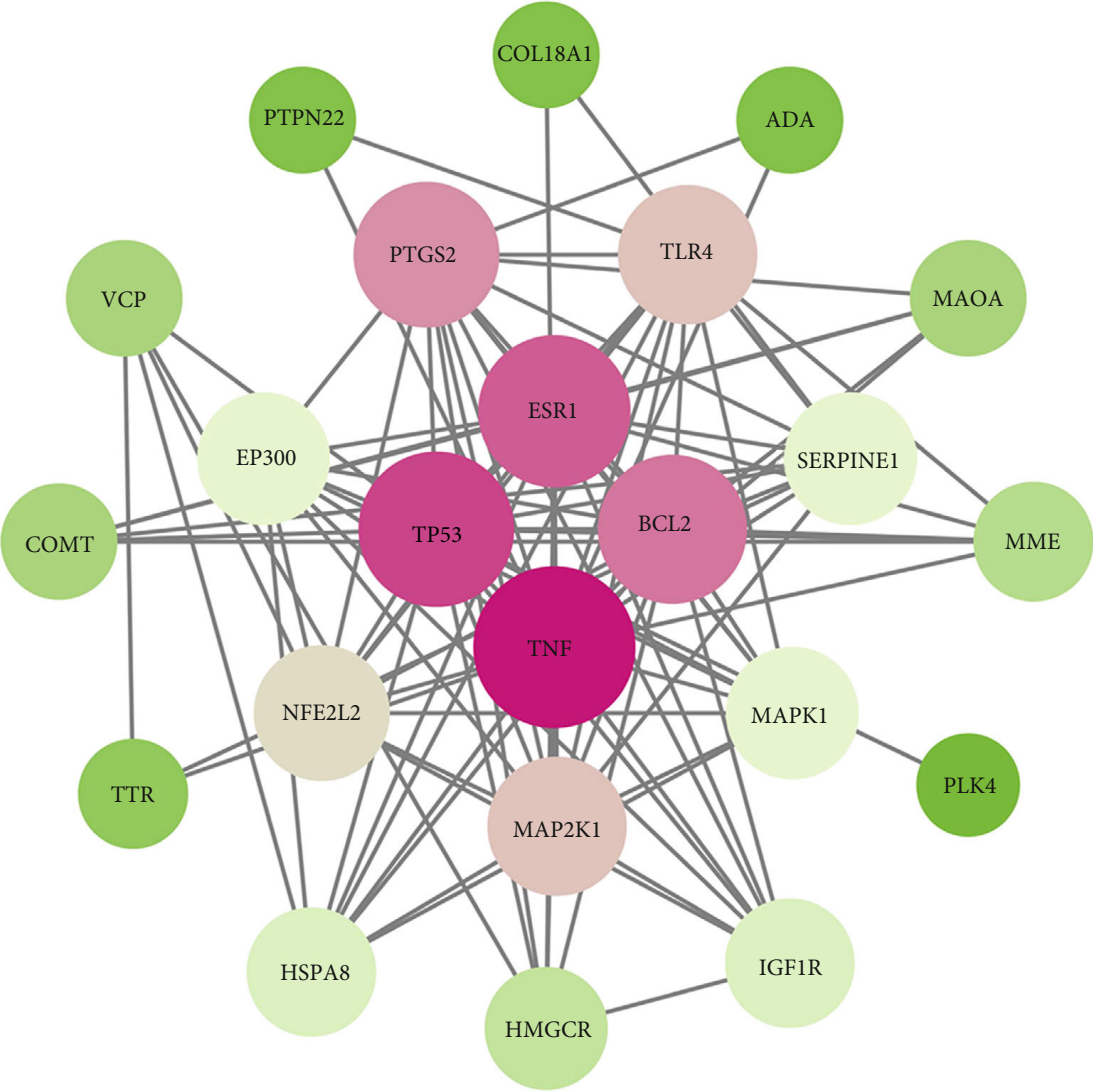
(c)

**Figure:**
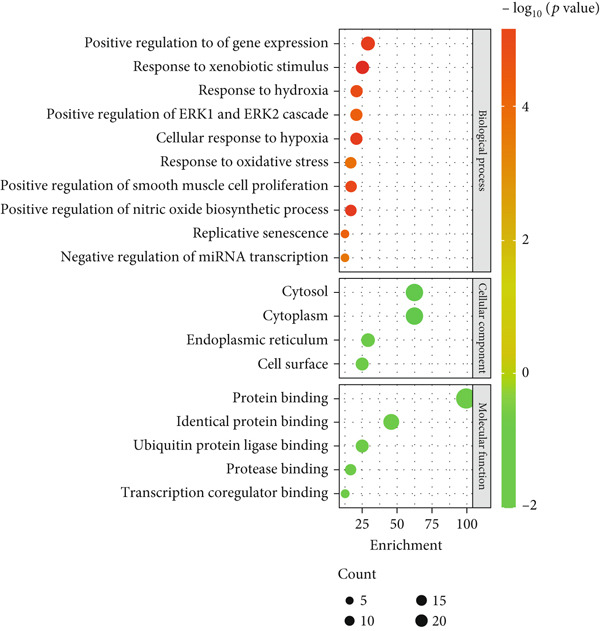
(d)

**Figure:**
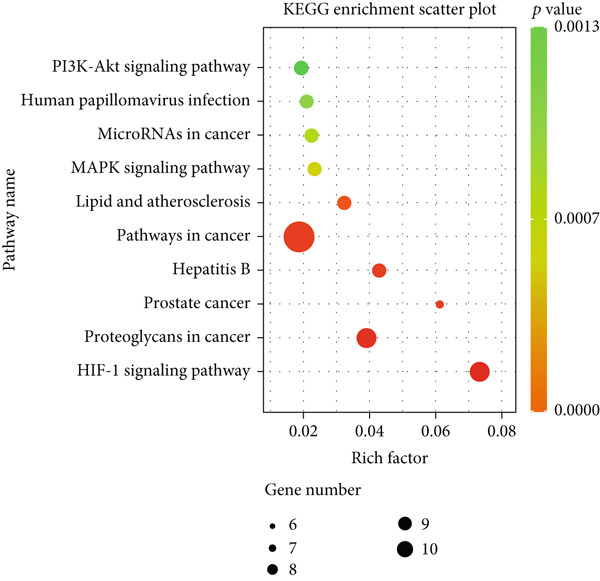
(e)

**Figure:**
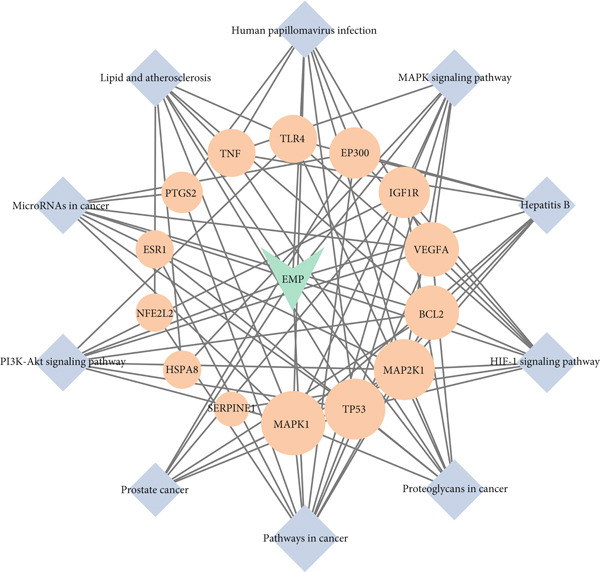
(f)

**Figure:**
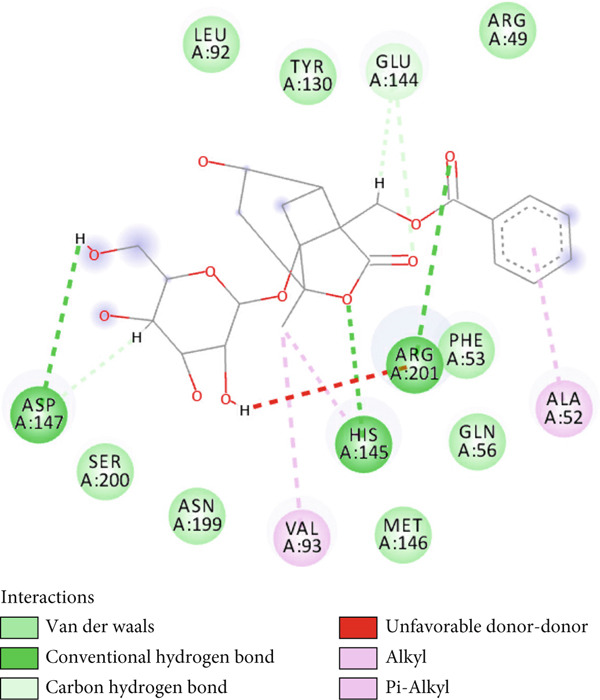
(g)

**Table 2:** Top 10 core targets.

**Targets**	**Degree**	**Betweenness centrality**	**Closeness centrality**	**Neighborhood connectivity**
TNF	19	0.241254684	0.88	9.052631579
TP53	17	0.158952066	0.814814815	10.05882353
ESR1	16	0.054574557	0.785714286	10.6875
BCL2	15	0.034324437	0.75862069	11.13333333
PTGS2	14	0.051602257	0.733333333	11.07142857
MAP2K1	12	0.006785542	0.6875	12.66666667
TLR4	12	0.035387893	0.6875	11.75
NFE2L2	11	0.017130388	0.666666667	12.63636364
MAPK1	10	0.001503127	0.647058824	13.5
SERPINE1	10	0.048182505	0.647058824	11.5

### 3.3. AF Improves HUA‐CI by Inhibiting the HIF‐1 Signaling Pathway

The hypoxia‐inducible factor‐1 (HIF‐1) signaling pathway plays a significant role in cognitive impairment [16]. Given that AF has been shown to alleviate cognitive disorders [17] and MAP2K1 regulates the HIF‐1*α* signaling pathway [18], while hyperuricemia activates the ERK pathway influencing HIF‐1*α* [19], we investigated whether AF improves HUA‐CI by modulating the HIF‐1 signaling pathway. We examined the effects of different concentrations of EMP on the HIF‐1 signaling pathway. The experiment included five groups: Control, HUA‐CI model, HUA‐CI + low dose EMP, HUA‐CI + medium dose EMP, and HUA‐CI + high dose EMP. Western blot analysis (Figure 4a) showed that compared with the Control group, HUA‐CI mice exhibited significantly increased expression of HIF‐1‐related proteins in the hippocampus, indicative of HIF‐1 pathway activation. However, in the HUA‐CI + EMP groups, the expression of these proteins decreased with increasing EMP concentration, suggesting a concentration‐dependent inhibition of the HIF‐1 signaling pathway by EMP. Further investigation into the mechanism of AF’s effect on the HIF‐1 signaling pathway involved three groups: HUA‐CI model, HUA‐CI + AF, and HUA‐CI + AF + FG4592 (HIF‐1*α* activator). Body weights remained stable without significant differences during the experimental period (Figure 4b). The results of Morris water maze experiment (Figure 4c) found that AF‐treated mice had shorter escape latency and enhanced spatial exploration ability. HE staining (Figure 4d) revealed that AF treatment alleviated hippocampal pathological damage in HUA‐CI mice. ELISA analysis of hippocampal inflammation (Figure 4e) showed that AF significantly reduced the levels of inflammatory cytokines (IL‐1*β*, IL‐6, and TNF‐*α*). Furthermore, ELISA analysis (Figure 4f) indicated that AF increased SOD and CAT activities and decreased MDA levels in the hippocampus, demonstrating its antioxidant effects. Flow cytometry (Figure 4g) showed that AF could reduce apoptosis in the hippocampus of HUA‐CI mice. Apoptosis was further increased after FG4592 was added. Finally, Western blot analysis (Figure 4h,i) was conducted to detect the expression of apoptosis‐related proteins Bax and Caspase‐3, as well as the inflammatory factor TNF‐*α*. The results showed that AF treatment reduced the expression levels of Bax, Caspase‐3, and TNF‐*α*. The addition of FG4592 further enhanced these effects, leading to a more pronounced decrease in the expression of these markers. In summary, our study demonstrates that AF can improve cognitive impairment and associated pathological changes caused by hyperuricemia by inhibiting the HIF‐1 signaling pathway, thereby reducing inflammation, lowering oxidative stress, and preventing apoptosis.

Figure 4AF improves HUA‐CI by inhibiting the HIF‐1 signaling pathway. (a) Western blot analysis. (b) Changes in body weight. (c) Morris water maze test. (d) HE staining. Scale bar: 50 *μ*m. (e) ELISA analysis measuring levels of inflammatory cytokines in hippocampal tissue. (f) ELISA analysis measuring oxidative stress markers in hippocampal tissue. (g) Flow cytometry assessing apoptosis in the hippocampal region. (h–i) Western blotting detecting expression of apoptosis‐related and inflammation‐related proteins in hippocampal tissue. *n* = 5. ∗*p* < 0.05, ∗∗*p* < 0.01, ∗∗∗*p* < 0.001.

**Figure:**
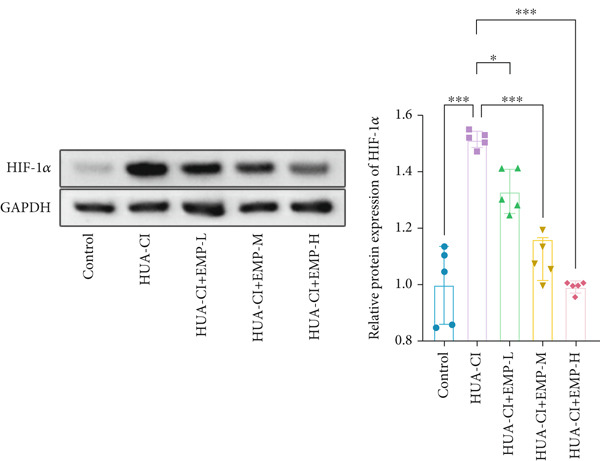
(a)

**Figure:**
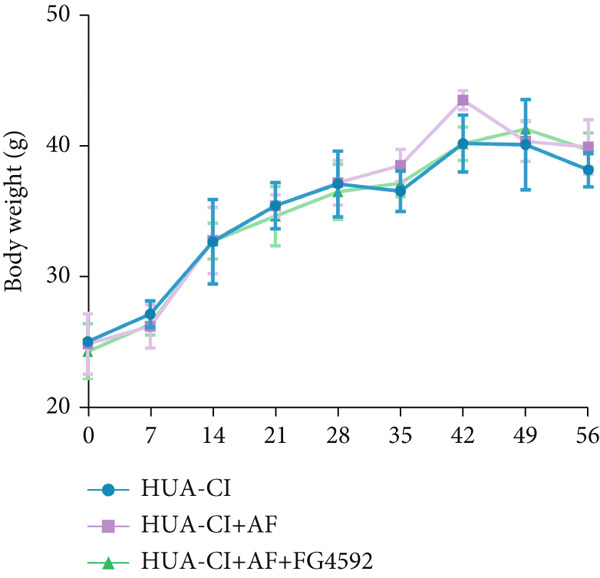
(b)

**Figure:**
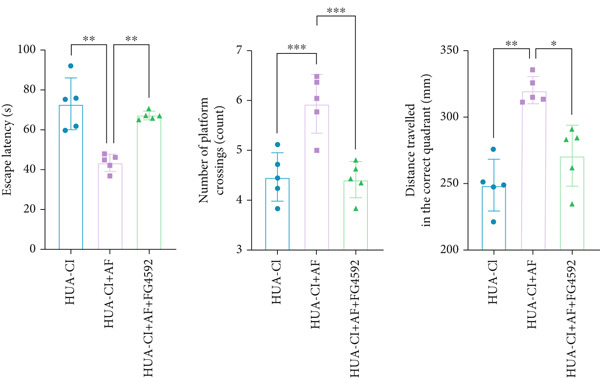
(c)

**Figure:**
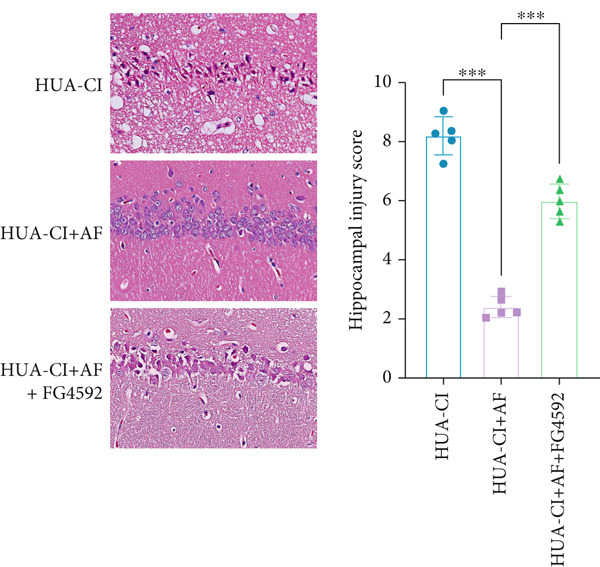
(d)

**Figure:**
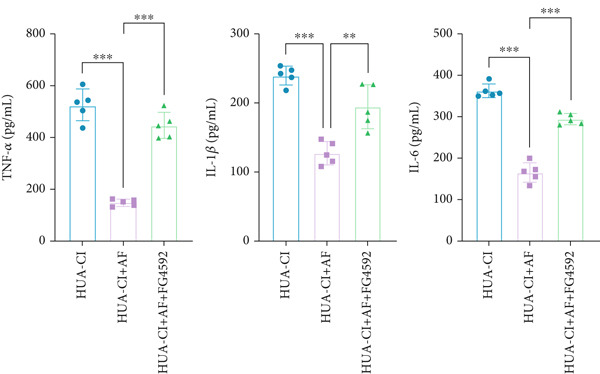
(e)

**Figure:**
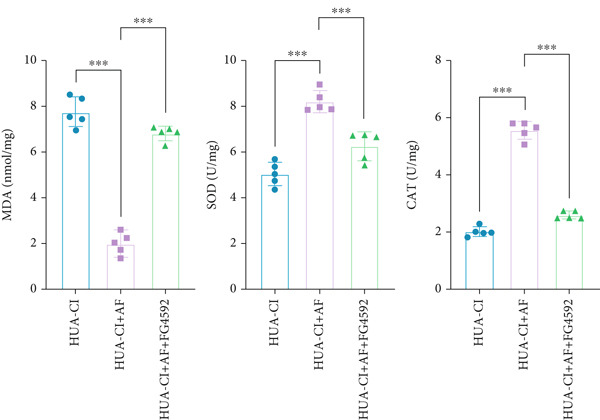
(f)

**Figure:**
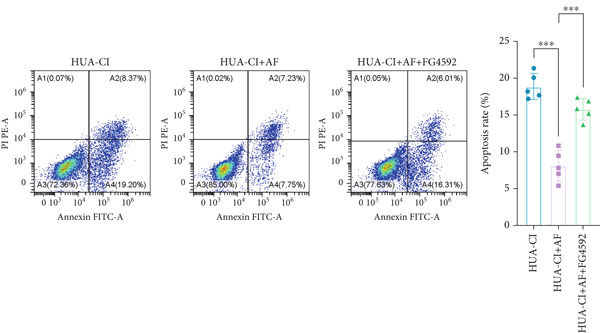
(g)

**Figure:**
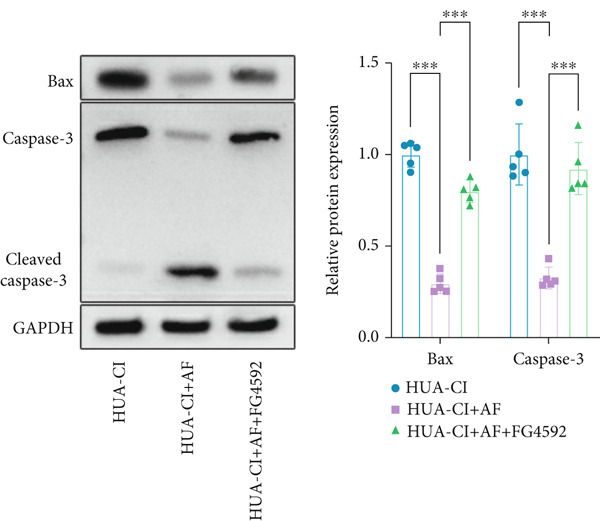
(h)

**Figure:**
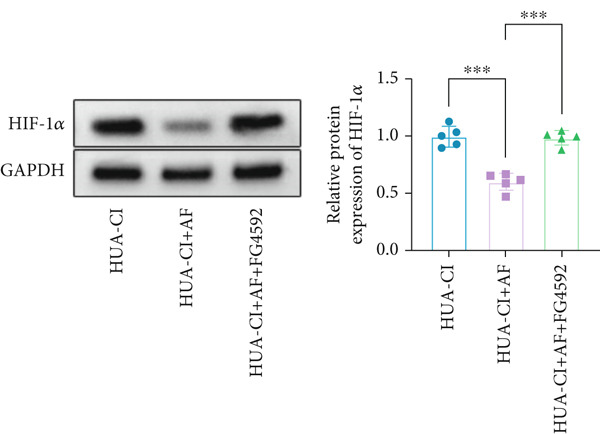
(i)

### 3.4. Effects of AF on Microglia Activated by UA Through HIF‐1 Signaling Pathway Activation

To elucidate the mechanisms by which AF exerts its effects in HUA‐CI, we established an in vitro model using UA‐treated microglial BV2 cells and evaluated the impact of AF on microglial activation, inflammatory response, and oxidative stress. The experiment included five groups: Control (no UA treatment), UA, UA + AF, UA + FG4592 (HIF‐1*α* activator), UA + AF + FG4592. Western blot analysis revealed upregulated expression of HIF‐1*α* in the UA group, which was significantly inhibited by AF treatment (Figure 5a). Cell viability, assessed by CCK‐8 assay, showed that UA treatment significantly reduced BV2 cell viability (Figure 5b). However, AF treatment partially restored cell viability, suggesting a protective role of AF against UA‐induced damage in microglia. PCR and Western blotting analyses demonstrated the influence of AF on microglial polarization (Figure 5c,d). In the HUA‐CI group, mRNA and protein expression of the M1 polarization marker CD86 increased, while the M2 polarization marker ARG1 decreased. AF treatment significantly downregulated CD86 expression and upregulated ARG1 expression, indicating a shift from pro‐inflammatory M1 to anti‐inflammatory M2 phenotype. ELISA results showed that levels of inflammatory cytokines IL‐1*β*, IL‐6, and TNF‐*α* in the supernatant of UA BV2 cells were significantly elevated (Figure 5e). AF treatment effectively reduced the secretion of these cytokines, demonstrating its anti‐inflammatory properties. Additionally, ELISA analysis (Figure 5f) revealed that AF treatment enhanced the activities of superoxide dismutase (SOD) and catalase (CAT) and decreased malondialdehyde (MDA) levels, confirming that AF strengthens cellular antioxidant defenses and mitigates oxidative stress damage. Collectively, our findings suggest that AF can protect microglia from HUA‐related injury by activating the HIF‐1 signaling pathway, thereby inhibiting UA‐induced microglial activation, reducing inflammation, and alleviating oxidative stress.

Figure 5Effects of AF on microglia activated by uric acid through activation of the HIF‐1 signaling pathway. (a) Western blot analysis. (B) CCK‐8 assay measuring cell viability. (c) PCR detecting mRNA expression of microglial activation markers. (d) Western blotting detecting protein expression of microglial activation markers. (e) ELISA analysis measuring levels of inflammatory cytokines in cell culture supernatants. (f) Analysis of MDA levels, SOD, and CAT activities (oxidative stress markers) in cell culture supernatants. *n* = 5. ∗*p* < 0.05, ∗∗*p* < 0.01, ∗∗∗*p* < 0.001.

**Figure:**
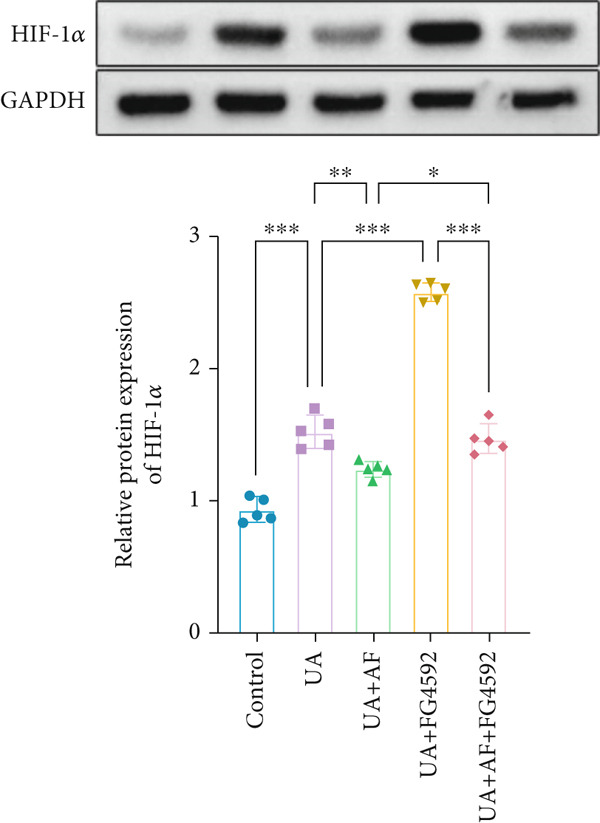
(a)

**Figure:**
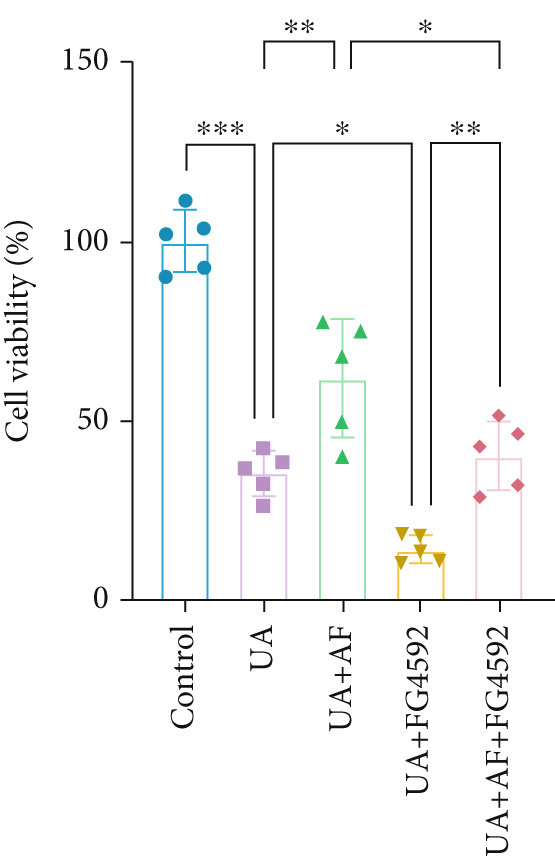
(b)

**Figure:**
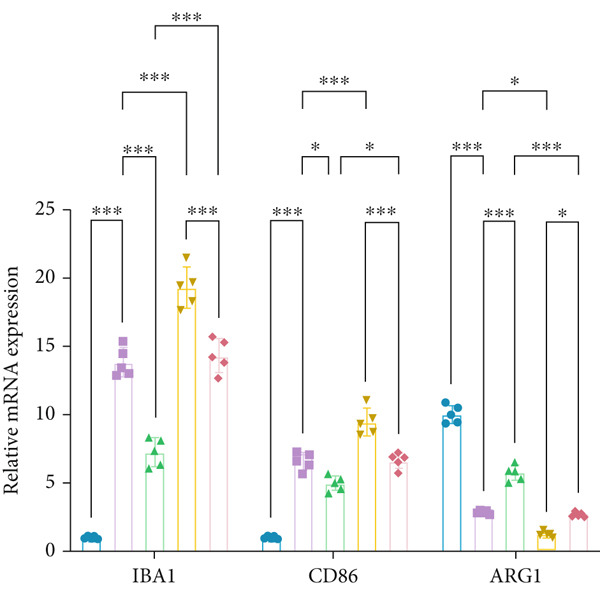
(c)

**Figure:**
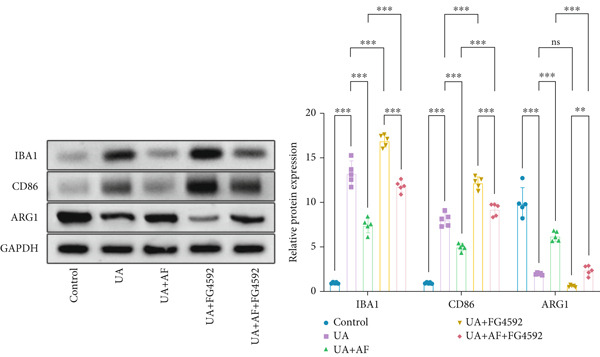
(d)

**Figure:**
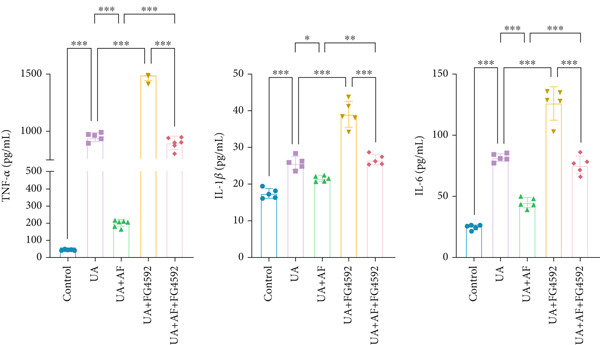
(e)

**Figure:**
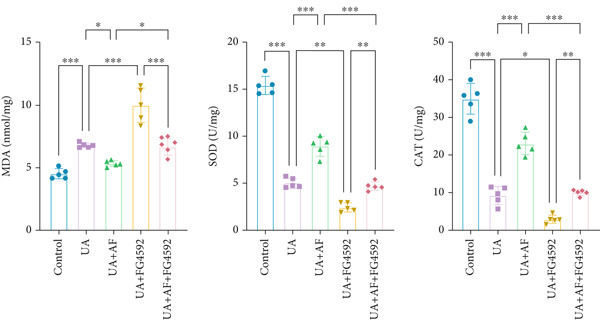
(f)

### 3.5. Protective Effects of AF on UA‐Induced Neuronal Apoptosis

To investigate the neuroprotective effects of AF in HUA‐CI, we utilized HT22 neuronal cells to evaluate the impact of AF on UA‐induced neuronal apoptosis. The experiment comprised five groups: Control (no UA treatment), UA, UA + AF, UA + FG4592 (HIF‐1*α* activator), UA + AF + FG4592. MTT assays showed that UA treatment significantly reduced HT22 neuronal cell viability (Figure 6a). AF treatment partially restored cell viability, indicating its potential to protect neurons from UA‐induced damage. The cell viability of UA + FG4592 group was the lowest, and the cell viability increased after adding AF, which further indicated that AF may protect neurons from UA‐induced damage. Flow cytometry and TUNEL staining were employed to assess apoptosis (Figure 6b,c). Results showed a significant increase in neuronal apoptosis in the UA group. AF treatment significantly reduced the number of TUNEL‐positive cells and the proportion of apoptotic cells (Figure 6b). The number of TUNEL‐positive cells in UA + FG4592 group was the highest, and the number of TUNEL‐positive cells decreased after adding AF. Flow cytometric analysis further confirmed that AF treatment markedly inhibited UA‐induced apoptosis (Figure 6c). Among them, the apoptosis rate of UA + FG4592 group was the highest, and the apoptosis rate was decreased after adding AF. Collectively, our study indicates that AF can protect neurons from HUA‐related damage by mitigating UA‐induced neuronal apoptosis.

Figure 6AF ameliorates UA‐induced neuronal apoptosis. (a) MTT assay measuring HT22 cell viability. (b) TUNEL staining assessing cell apoptosis. Scale bar: 50 *μ*m. (c) Flow cytometry analyzing cell apoptosis. *n* = 5. ∗*p* < 0.05, ∗∗*p* < 0.01, ∗∗∗*p* < 0.001.

**Figure:**
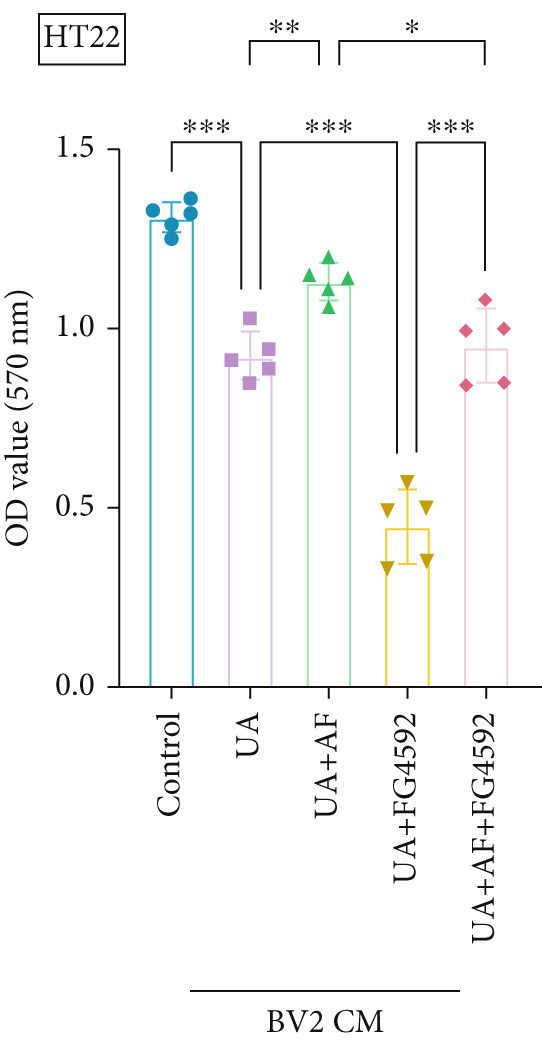
(a)

**Figure:**
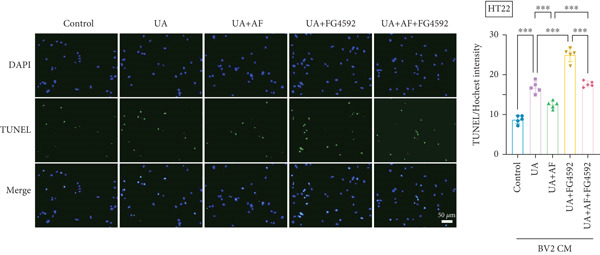
(b)

**Figure:**
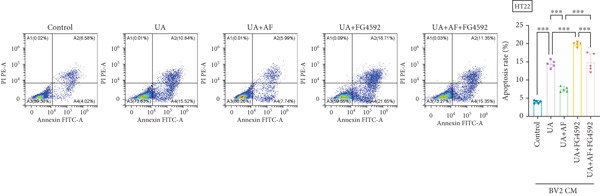
(c)

### 3.6. Mechanisms of AF’s Effects on Microglia and Neurons Via Targeting MAP2K1

Based on the molecular docking results showing that AF can bind to MAP2K1, we used CETSA to verify this interaction in BV2 cells. The results indicated that AF binding increased the melting temperature of MAP2K1, thereby inhibiting its thermal denaturation (Figure 7a). In addition, AF treatment of BV2 cells, compared with DMSO treatment, significantly downregulated MAP2K1 mRNA and protein levels (Figure 7b,c). Therefore, AF can bind to MAP2K1 and affect its expression and activity.

Figure 7Effects of AF on microglia and neurons by targeting MAP2K1 to inhibit the HIF‐1 signaling pathway. (a) CETSA, representative Western blot images of MAP2K1 protein levels, treated or untreated with AF, evaluated after incubation at different temperature points for 5 min. (b) PCR detecting mRNA expression of MAP2K1. (c) Western blot analysis of MAP2K1. (d) Western blot analysis of MAP2K1, p‐ERK, and HIF‐1*α* expression. (e) CCK‐8 assay measuring cell viability. (f) Western blotting detecting protein expression of microglial activation markers. (g) ELISA analysis measuring levels of inflammatory cytokines in cell culture supernatants. (h) ELISA analysis measuring levels/activities of SOD, CAT, and MDA in cells. (i) MTT assay measuring HT22 cell viability. (j) Flow cytometry analyzing cell apoptosis. (k) TUNEL staining assessing cell apoptosis. Scale bar: 50 *μ*m. *n* = 5. ∗*p* < 0.05, ∗∗*p* < 0.01, ∗∗∗*p* < 0.001.

**Figure:**
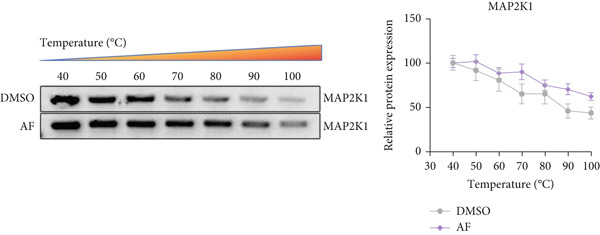
(a)

**Figure:**
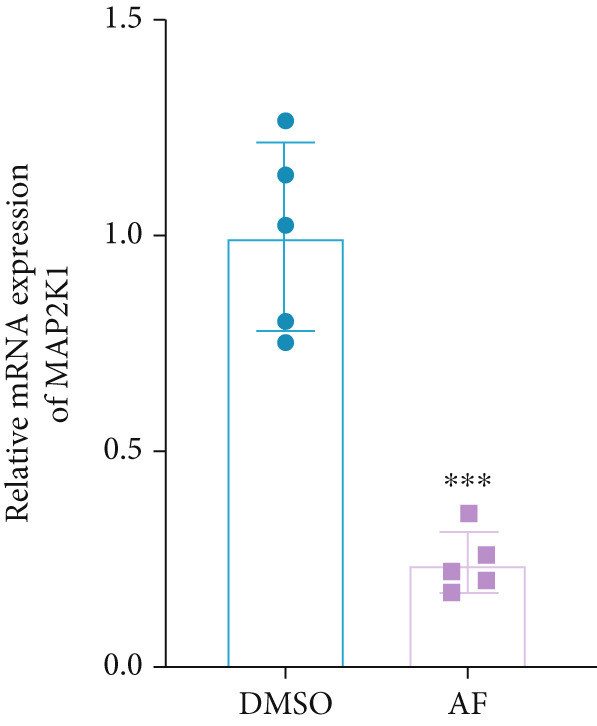
(b)

**Figure:**
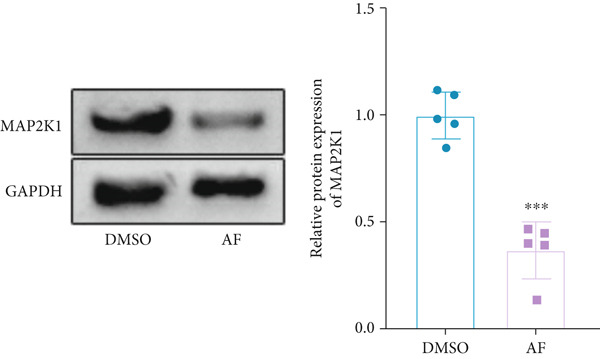
(c)

**Figure:**
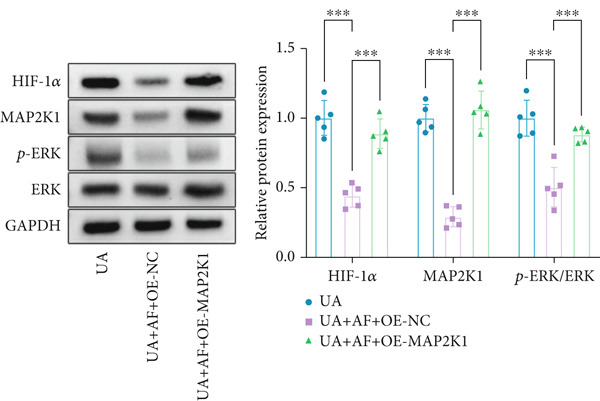
(d)

**Figure:**
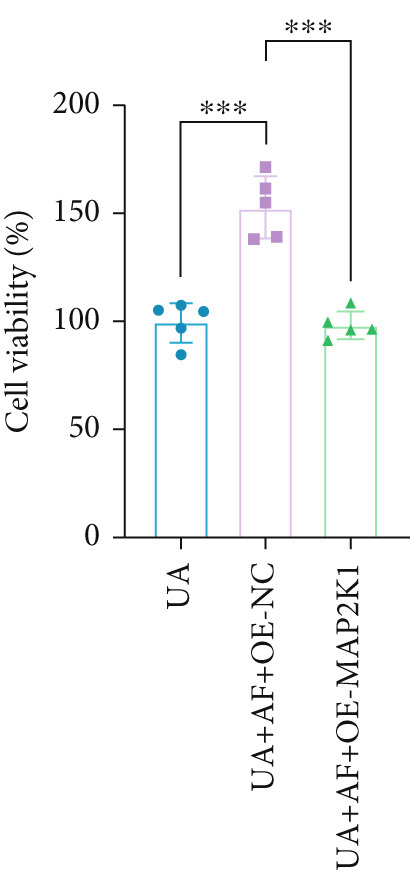
(e)

**Figure:**
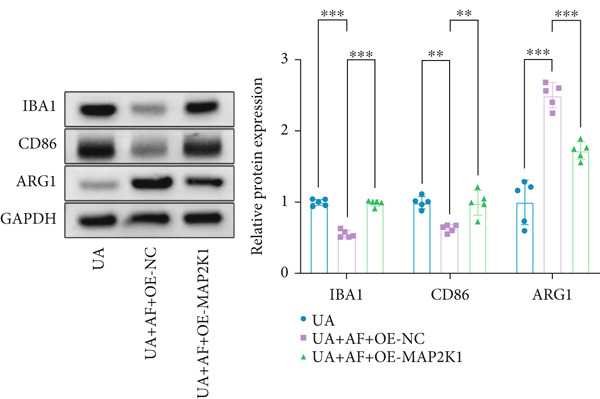
(f)

**Figure:**
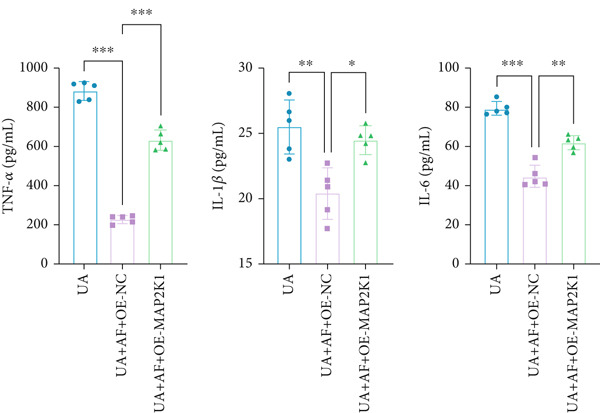
(g)

**Figure:**
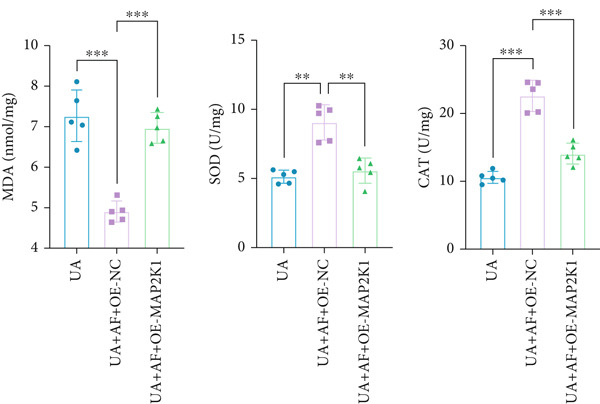
(h)

**Figure:**
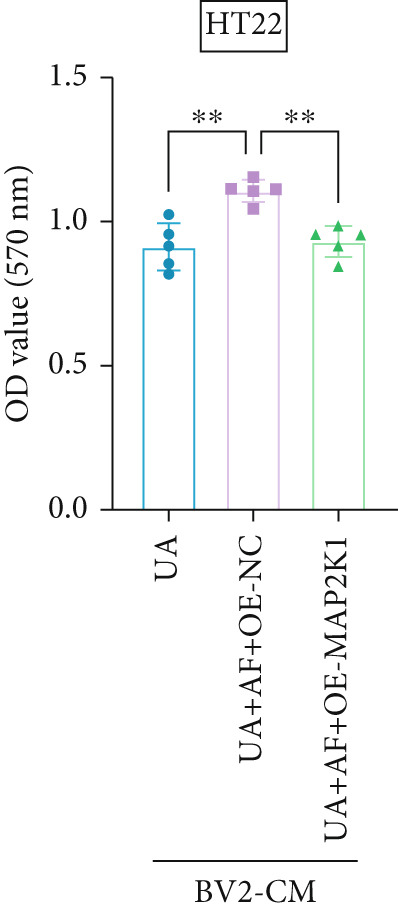
(i)

**Figure:**
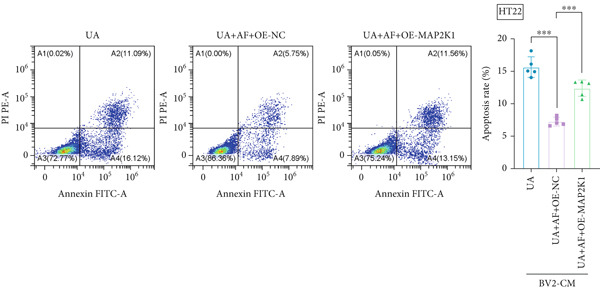
(j)

**Figure:**
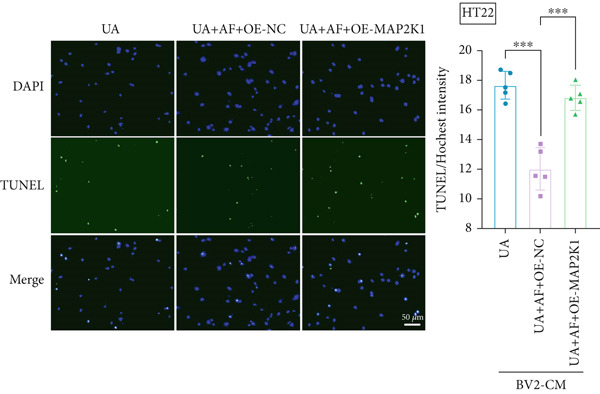
(k)

To gain deeper insights into the specific molecular mechanisms of AF in HUA‐CI, we investigated whether AF targets MAP2K1 (MEK) to inhibit the HIF‐1 signaling pathway, thereby affecting microglial and neuronal functions. The experiment included three groups: UA (uric acid‐treated), UA + AF + OE‐NC (overexpression negative control), and UA + AF + OE‐MAP2K1 (MAP2K1 overexpression). Western blot analysis showed that AF treatment significantly inhibited the expression of MAP2K1 and its downstream molecules such as p‐ERK and HIF‐1*α* proteins. Overexpression of MAP2K1 weakened the protective effect of AF, suggesting that AF regulates the HIF‐1 signaling pathway by targeting MAP2K1 (Figure 7d). CCK‐8 detection (Figure 7e) showed that AF treatment partially restored the vitality of BV2 microglia after UA treatment, while MAP2K1 overexpression weakened this protective effect, further confirming that the effect of AF depends on the regulation of MAP2K1. Western blotting analysis revealed the impact of AF on microglial polarization (Figure 7f). In the UA treatment group, the protein expression of M1‐type polarization marker CD86 was at a high level, while the expression of M2‐type polarization marker ARG1 was at a low level. AF treatment significantly downregulated CD86 expression and upregulated ARG1 expression, which promoted a shift from M1 to M2 phenotype. This effect was reversed when MAP2K1 was overexpressed. ELISA detection showed that the levels of inflammatory factors IL‐1*β*, IL‐6, and TNF‐*α* in UA‐treated BV2 cell culture supernatant were in a high state (Figure 7g). AF treatment effectively reduced the secretion of these cytokines, but this effect was weakened by MAP2K1 overexpression. ELISA analysis demonstrated that AF treatment enhanced SOD and CAT activities and decreased MDA levels (Figure 7h), indicating strengthened antioxidant defense and reduced oxidative stress damage. However, MAP2K1 overexpression diminished these effects. For HT22 neuronal cells, the results of MTT detection (Figure 7i) showed that AF treatment could partially restore the vitality of HT22 cells of neurons, suggesting that AF may protect neurons from UA‐induced damage. However, this protective effect was weakened when MAP2K1 was overexpressed. Flow cytometry and TUNEL staining were used to assess apoptosis (Figure 7j,k). Results showed AF treatment significantly reduced the number of TUNEL‐positive cells and the proportion of apoptotic cells (Figure 7k). Flow cytometric analysis further confirmed that AF treatment markedly inhibited UA‐induced apoptosis (Figure 7j). However, MAP2K1 overexpression weakened AF’s anti‐apoptotic effect. In summary, our study demonstrates that AF can protect microglia and neurons from HUA‐related damage by targeting MAP2K1 to inhibit the HIF‐1 signaling pathway, thereby reducing inflammation, lowering oxidative stress, and inhibiting apoptosis.

## 4. Discussion

This study systematically investigated the therapeutic effects and mechanisms of EMP and its primary active component, AF, on HUA‐CI through a combination of network pharmacology and experimental validation. Our findings demonstrate that EMP significantly improves cognitive function in HUA‐CI mice, mitigates hippocampal pathological damage, and exerts protective effects by suppressing inflammatory responses, reducing oxidative stress, and preventing apoptosis. Research by Xu et al. has shown that active components from *Phellodendron amurense* can lower hyperuricemia via multiple mechanisms, including influencing purine metabolism and inhibiting the expression of XOD and ABCG2 [20]. Various flavonoids, such as those extracted from safflower and *Plumeria rubra* flowers, have also demonstrated potential in inhibiting hyperuricemia [21]. *Selaginella moellendorffii* has been found to significantly reduce serum UA levels and decrease the expression of inflammatory factors in the synovial fluid of hyperuricemic mice [22]. In comparison with these studies, our research highlights the multifaceted roles of EMP and AF in treating HUA‐CI, aligning with previous findings on TCM components and formulations, which underscore the potential value and multiplicity of TCM in addressing hyperuricemia and associated conditions.

Network pharmacology analysis revealed that EMP exerts its therapeutic effects on HUA‐CI through multi‐target and multi‐pathway mechanisms, particularly emphasizing the strong binding affinity between AF and MAP2K1. Previous studies have indicated that the HIF‐1 signaling pathway plays a critical role in cognitive disorders [16], and AF has been shown to alleviate cognitive impairments [17]. Additionally, MAP2K1 regulates the HIF‐1*α* signaling pathway [18], while hyperuricemia activates the ERK pathway, further impacting HIF‐1*α* [19]. Based on this background, we explored whether AF could improve HUA‐CI by modulating the HIF‐1 signaling pathway. Our results indicate that AF dose‐dependently inhibits the HIF‐1 signaling pathway, leading to significant improvements in behavioral performance, reduction in pathological damage, and inhibition of apoptosis in HUA‐CI mice. Park et al. found that HIF‐1*α* activity is regulated by various factors. Under normoxic conditions, HIF‐1*α* is hydroxylated by prolyl hydroxylase domain‐containing proteins (PHDs) and subsequently ubiquitinated and degraded [23]. However, under hypoxic conditions, PHD becomes inactive, preventing the degradation of HIF‐1*α*, thus forming an active HIF‐1 heterodimer. This suggests that in the context of hyperuricemia, characterized by oxidative stress and metabolic abnormalities, the stability and activity of HIF‐1*α* may be compromised. Zhang et al. discovered that HIF‐1*α* exerts multiple biological effects within cells, including promoting angiogenesis, regulating energy metabolism, and responding to hypoxic environments [24]. In hyperuricemia, the reduction in HIF‐1*α* may lead to abnormalities in these physiological processes, thereby affecting cardiovascular health. Other studies [25, 26] have shown that inhibiting HIF‐1*α* can significantly reduce the expression and secretion of inflammatory cytokines such as IL‐1*β* and TNF‐*α* under high‐glucose conditions. This indicates that inhibiting HIF‐1*α* might help mitigate inflammation and cellular damage caused by hyperuricemia. In summary, our study provides comprehensive insights into the mechanisms by which EMP and AF exert their therapeutic effects on HUA‐CI, highlighting the potential of TCM in managing hyperuricemia and related disorders. The findings support the development of new strategies targeting HUA‐CI and other complex diseases characterized by multiple pathophysiological processes.

Our study revealed that AF mitigates UA‐induced microglial activation and the associated inflammatory response by inhibiting the HIF‐1 signaling pathway, while enhancing the cellular antioxidant defense system. Microglial activation is a significant source of neuroinflammation, and the anti‐inflammatory and antioxidant effects of AF suggest its potential for broad‐spectrum neuroprotection. This finding extends the pharmacological scope of AF and provides a novel therapeutic target for treating neuroinflammation. Other phytochemicals, such as polyphenols and paeoniflorin, modulate inflammatory responses through various signaling pathways, including TLR4/NF‐*κ*B and PINK1/BAD [27, 28]. Polyphenolic phytochemicals exert pharmacological effects on intestinal inflammation by targeting the TLR4/NF‐*κ*B pathway [26], indicating that these compounds may inhibit inflammatory responses via modulation of specific signaling pathways. Paeoniflorin has been shown to alleviate dextran sulfate sodium‐induced colitis through a TLR4‐dependent mechanism, demonstrating its anti‐inflammatory properties [28]. Additionally, paeoniflorin has been found to protect spiral ganglion neurons from cisplatin‐induced ototoxicity, potentially involving the PINK1/BAD pathway [28]. In this study, we focused on the role of AF in UA‐induced microglial activation and inflammation, suggesting that its anti‐inflammatory and immunomodulatory effects may contribute to its neuroprotective actions. This provides scientific evidence for developing new therapeutic strategies.

Notably, EMP/AF’s anti‐inflammatory effects in HUA‐CI may involve crosstalk with other key pathways regulating microglial activation. AF has been shown to suppress inflammation via inhibiting NF‐*κ*B/NLRP3 and PI3K/AKT pathways in other disease models, and to alleviate neuroinflammation through the PGK1/Nrf2/HO‐1 pathway [29–31]. Importantly, NF‐*κ*B, PI3K/AKT, and Nrf2 pathways are critical for mitigating hyperuricemia‐related inflammation [32, 33], and their dysregulation directly promotes microglial activation [34–36]. These findings collectively suggest that AF could potentially modulate these pathways to inhibit microglial activation in HUA‐CI. However, direct evidence that AF regulates NF‐*κ*B, PI3K/AKT, or Nrf2 signaling in hyperuricemia‐induced microglial inflammation is currently lacking. Future studies focusing on this specific context would help clarify the multi‐pathway coordination underlying EMP/AF’s anti‐inflammatory effects.

Furthermore, our research demonstrated that AF significantly protects HT22 neuronal cells from UA‐induced apoptosis, restoring cell viability. Neuronal apoptosis is a critical factor contributing to cognitive impairment in hyperuricemia [37], and the anti‐apoptotic effects of AF offer a new approach for prevention and treatment. This finding further supports the potential of AF in neuroprotection. Similar neuroprotective effects have been observed with other plant‐derived components. For example, plant polyphenols such as sulforaphane and resveratrol protect neurons from oxidative stress and inflammatory damage by activating the Nrf2 antioxidant pathway, thereby improving pathological conditions in neurodegenerative diseases [38, 39]. Other studies [40, 41] have found that plant extracts like ginsenoside A, baicalin, and kaempferol exhibit anti‐inflammatory, antioxidant, and neuroprotective effects by modulating neural signaling pathways such as GABA receptors and P2X3 receptors, alleviating neuropathic pain and promoting neurovascular function recovery.

Moreover, our study uncovered that AF significantly improves the function of microglia and neurons by targeting MAP2K1 to inhibit the HIF‐1 signaling pathway, reducing inflammation and oxidative stress, and inhibiting apoptosis. As a key node connecting multiple signaling pathways, MAP2K1’s regulation by AF showcases its multipotent therapeutic potential. Our findings not only elucidate the specific mechanisms of AF but also provide important references for future drug development targeting MAP2K1. Studies by Yang et al. showed that Danggui Shaoyao San (DSS) enhances A*β* clearance by increasing Trem2 expression and promoting M1‐to‐M2 polarization of microglia, thereby alleviating inflammation [40]. Chan et al. demonstrated that ESEAF, a plant extract, suppresses inflammatory responses and reduces microglial activation, mitigating neuroinflammation [42]. These studies further illustrate that plant extracts may improve microglial function by inhibiting inflammatory signaling pathways.

In summary, our study combined network pharmacology and experimental validation to reveal the multifaceted roles and underlying mechanisms of EMP and its primary active component AF in treating HUA‐CI. The findings highlight the potential of EMP as a multi‐target therapeutic strategy and underscore the critical role of AF in regulating the HIF‐1 signaling pathway and protecting neural cells. Despite these insights, limitations of our study include a limited sample size and the lack of long‐term clinical data, which should be addressed in future investigations.

## Conflicts of Interest

The authors declare no conflicts of interest.

## Funding

This study was supported by Social Development of Shaanxi Province Key Project, No. 2018SF‐315; Project of Shaanxi Provincial Key Laboratory of Biomedicine, No. 2018SZS41; the Shaanxi Province Key Research and Development Plan Projects, No. 2023‐YBSF‐566; the Shaanxi Fengdan Zhengyuan Biotechnology Limited Company.

## Supporting information


**Supporting Information** Additional supporting information can be found online in the Supporting Information section. Supporting Figure S1: Drug‐active component‐target network. Supporting Figure S2: The “component–target–pathway” relationship network diagram. Supporting Table S1: Contents of the 21 components of EMP across 10 batches.

## Data Availability

The data that are available from the corresponding author upon reasonable request.
