# Supplementary material for: Albiflorin‐Mediated MAP2K1 Targeting and HIF‐1 Signaling Inhibition Contribute to the Therapeutic Efficacy in Hyperuricemia‐Associated Cognitive Impairment
Source: Hum Mutat. 2026 Jan 19;2026:5859468. doi: 10.1155/humu/5859468 (PMC12813877; doi:10.1155/humu/5859468)
Supplement: Supplementary file 1 — Supporting Information Additional supporting information can be found online in the Supporting Information section. Supporting Figure S1: Drug‐active component‐target network. Supporting Figure S2: The “component–target–pathway” relationship network diagram. Supporting Table S1: Contents of the 21 components of EMP across 10 batches. [file HUMU-2026-5859468-s001.pdf]

Supplementary Table S1. Contents of the 21 components of EMP across 10 batches (n = 3, mean ± SD, $mg.g^{-1}$).

Component	S1	S2	S3	S4	S5	S6	S7	S8	S9	S10	Mean
Vitamin B6	2.4589 ± 0.0079	2.4568 ± 0.0125	2.4591 ± 0.0102	2.4640 ± 0.0316	2.4602 ± 0.0118	2.4812 ± 0.0098	2.4671 ± 0.0192	2.4589 ± 0.0086	2.4590 ± 0.0154	2.4590 ± 0.0067	2.4624 ± 0.0073
Gallic acid	67.3194 ± 0.2817	67.1276 ± 0.3572	67.3418 ± 0.2186	67.3183 ± 0.3105	67.2277 ± 0.2418	67.3007 ± 0.1187	67.1792 ± 0.2943	67.1583 ± 0.3342	67.3219 ± 0.3562	67.3347 ± 0.2852	67.2630 ± 0.0817
Pyrocatechol	0.2660 ± 0.0017	0.2663 ± 0.0061	0.2652 ± 0.0034	0.2684 ± 0.0015	0.2654 ± 0.0071	0.2648 ± 0.0097	0.2658 ± 0.0056	0.2693 ± 0.0012	0.2689 ± 0.0089	0.2647 ± 0.0042	0.2665 ± 0.0017
Methyl gallate	0.7960 ± 0.0021	0.7943 ± 0.0018	0.7967 ± 0.0083	0.7952 ± 0.0017	0.7977 ± 0.0059	0.7911 ± 0.0076	0.7943 ± 0.0038	0.7953 ± 0.0076	0.7966 ± 0.0085	0.7984 ± 0.0028	0.7956 ± 0.0021
Hydroxypaeoniflorin	1.0150 ± 0.0018	1.0144 ± 0.0024	1.0149 ± 0.0032	1.0145 ± 0.0043	1.0152 ± 0.0099	1.0192 ± 0.0047	1.0176 ± 0.0052	1.0141 ± 0.0014	1.0151 ± 0.0061	1.0134 ± 0.0027	1.0153 ± 0.0018
Aesculetin	4.9691 ± 0.0053	4.9672 ± 0.0108	4.9687 ± 0.0096	4.9677 ± 0.0112	4.9690 ± 0.0078	4.9854 ± 0.0084	4.9694 ± 0.0073	4.9698 ± 0.0181	4.9695 ± 0.0057	4.9696 ± 0.0049	4.9705 ± 0.0053
Caffeic acid	0.5279 ± 0.0008	0.5284 ± 0.0006	0.5292 ± 0.0012	0.5280 ± 0.0016	0.5263 ± 0.0034	0.5276 ± 0.0016	0.5273 ± 0.0024	0.5274 ± 0.0015	0.5279 ± 0.0013	0.5269 ± 0.0026	0.5277 ± 0.0008
Albiflorin	4.6362 ± 0.0110	4.6352 ± 0.0145	4.6355 ± 0.0176	4.6367 ± 0.0128	4.6377 ± 0.0216	4.6359 ± 0.0156	4.6363 ± 0.0097	4.6361 ± 0.0119	4.6373 ± 0.0065	4.6383 ± 0.0078	4.6365 ± 0.0010
Procyanidine	2.3297 ± 0.0032	2.3283 ± 0.0106	2.3296 ± 0.0145	2.3298 ± 0.0098	2.3292 ± 0.0112	2.3296 ± 0.0078	2.3293 ± 0.0046	2.3277 ± 0.0051	2.3282 ± 0.0101	2.3389 ± 0.0023	2.3300 ± 0.0032
Paeoniflorin	8.9277 ± 0.0256	8.9213 ± 0.1262	8.9267 ± 0.0897	8.9246 ± 0.0573	8.9279 ± 0.0789	8.9264 ± 0.0923	8.9332 ± 0.0854	8.9421 ± 0.0842	8.9278 ± 0.0957	8.9281 ± 0.1106	8.9286 ± 0.0056
p-coumaric acid	0.3401 ±	0.3431 ±	0.3385 ±	0.3405 ±	0.3402 ±	0.3401 ±	0.3491 ±	0.3414 ±	0.3422 ±	0.3406 ±	0.3416 ±

	0.0029	0.0046	0.0032	0.0019	0.0015	0.0078	0.0091	0.0083	0.0076	0.0095	0.0029
Benzoic acid	7.1773 ± 0.0114	7.1769 ± 0.0342	7.1774 ± 0.0154	7.1779 ± 0.0263	7.1773 ± 0.0372	7.1792 ± 0.0712	7.1762 ± 0.0189	7.1802 ± 0.0664	7.1764 ± 0.0818	7.1800 ± 0.0471	7.1779 ± 0.0014
Ferulic acid	0.1419 ± 0.0014	0.1413 ± 0.0012	0.1423 ± 0.0008	0.1436 ± 0.0012	0.1433 ± 0.0016	0.1401 ± 0.0018	0.1453 ± 0.0015	0.1418 ± 0.0009	0.1422 ± 0.0011	0.1434 ± 0.0013	0.1425 ± 0.0014
2,4-dihydroxyacetophenone	0.2998 ± 0.0030	0.2995 ± 0.0023	0.2994 ± 0.0041	0.2976 ± 0.0023	0.2982 ± 0.0019	0.2988 ± 0.0024	0.2955 ± 0.0035	0.2997 ± 0.0029	0.2913 ± 0.0021	0.2933 ± 0.0027	0.2973 ± 0.0030
Gallogen	4.7710 ± 0.0154	4.7684 ± 0.0142	4.7754 ± 0.0217	4.7582 ± 0.0418	4.7723 ± 0.0424	4.7717 ± 0.0138	4.7710 ± 0.0287	4.7708 ± 0.0216	4.7754 ± 0.0487	4.7777 ± 0.0491	4.7712 ± 0.0054
1,2,3,6-tetra-O-galloyl-β-D-glucose	1.2149 ± 0.0047	1.2126 ± 0.0039	1.2159 ± 0.0096	1.2283 ± 0.0086	1.2143 ± 0.0071	1.2167 ± 0.0065	1.2143 ± 0.0078	1.2185 ± 0.0048	1.2219 ± 0.0035	1.2146 ± 0.0053	1.2172 ± 0.0047
1,2,3,4,6-O-pentagalloyl glucose	20.9119 ± 0.1156	20.9107 ± 0.1254	20.9129 ± 0.1089	20.9122 ± 0.1076	20.9385 ± 0.1247	20.9517 ± 0.1328	20.9216 ± 0.1423	20.9319 ± 0.1157	20.9119 ± 0.1125	20.9005 ± 0.1327	20.9204 ± 0.0156
Apigenin 7-O-neohesperidoside	0.4480 ± 0.0025	0.4481 ± 0.0013	0.4467 ± 0.0008	0.4481 ± 0.0011	0.4491 ± 0.0023	0.4438 ± 0.0019	0.4471 ± 0.0054	0.4470 ± 0.0023	0.4483 ± 0.0039	0.4410 ± 0.0033	0.4467 ± 0.0025
Apigenin-7-O-glucoside	0.6035 ± 0.0012	0.6041 ± 0.0009	0.6037 ± 0.0098	0.6035 ± 0.0054	0.6037 ± 0.0032	0.6049 ± 0.0075	0.6045 ± 0.0086	0.6056 ± 0.0035	0.6072 ± 0.0042	0.6050 ± 0.0054	0.6046 ± 0.0012
Mudanpioside C	0.3333 ± 0.0011	0.3349 ± 0.0014	0.3322 ± 0.0019	0.3348 ± 0.0022	0.3337 ± 0.038	0.3358 ± 0.0031	0.3342 ± 0.0037	0.3352 ± 0.0029	0.3355 ± 0.0026	0.3339 ± 0.0024	0.3343 ± 0.0011
Paeonol	0.1288 ± 0.0009	0.1291 ± 0.0004	0.1284 ± 0.0002	0.1290 ± 0.0015	0.1291 ± 0.0001	0.1278 ± 0.0007	0.1265 ± 0.0016	0.1299 ± 0.0003	0.1289 ± 0.0023	0.1289 ± 0.0024	0.1286 ± 0.0009

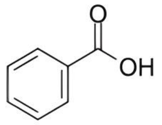

Benzoic acid (BA)

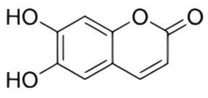

Aesculetin (AN)

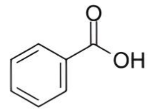

Pyrocatechol(PC)

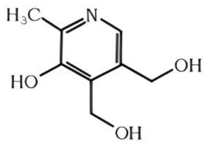

Vitamin B6 (VB)

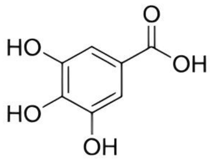

Gallic acid (GA)

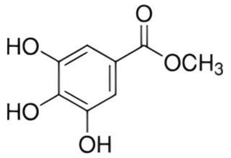

Methyl gallate (MG)

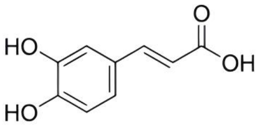

Caffeic acid (CA)

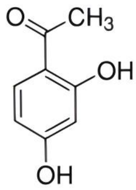

2,4-dihydroxy-acetophenone (DP)

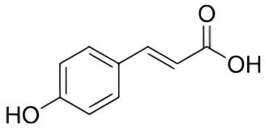

p-coumaric acid (PA)

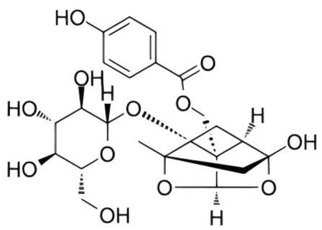

Hydroxypaeoniflorin (HP)

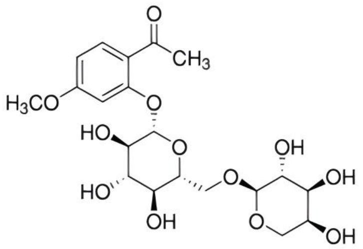

Alibiflorin(AF)

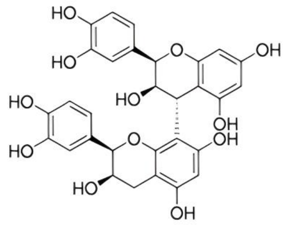

Proanthocyanidins B2 (PB)

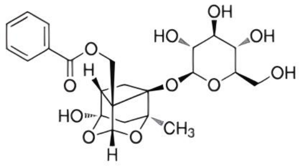

Paeoniflorin (PN)

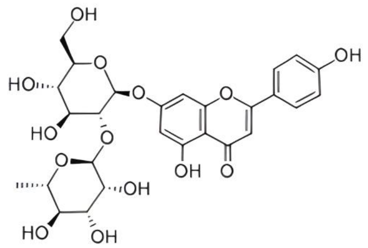

Apigenin 7-O-neohesperidoside (NP)

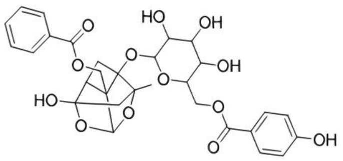

Moutonin C (MC)

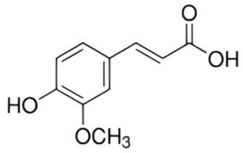

Ferulic acid (FA)

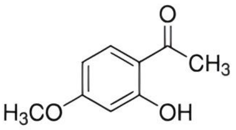

paeonol (PL)

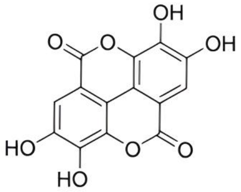

gallogen (GL)

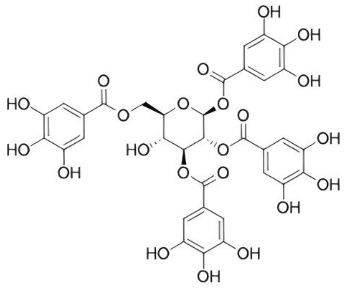

1,2,3,6-tetra-O-galloyl-β-D-glucose (GG)

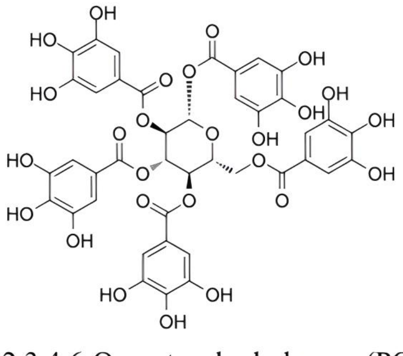

1,2,3,4,6-O-pentagaloyl glucose (PG)

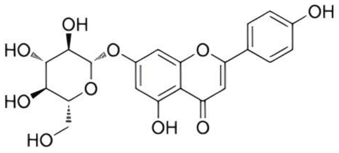

apigenin 7-O-glucoside (AG)

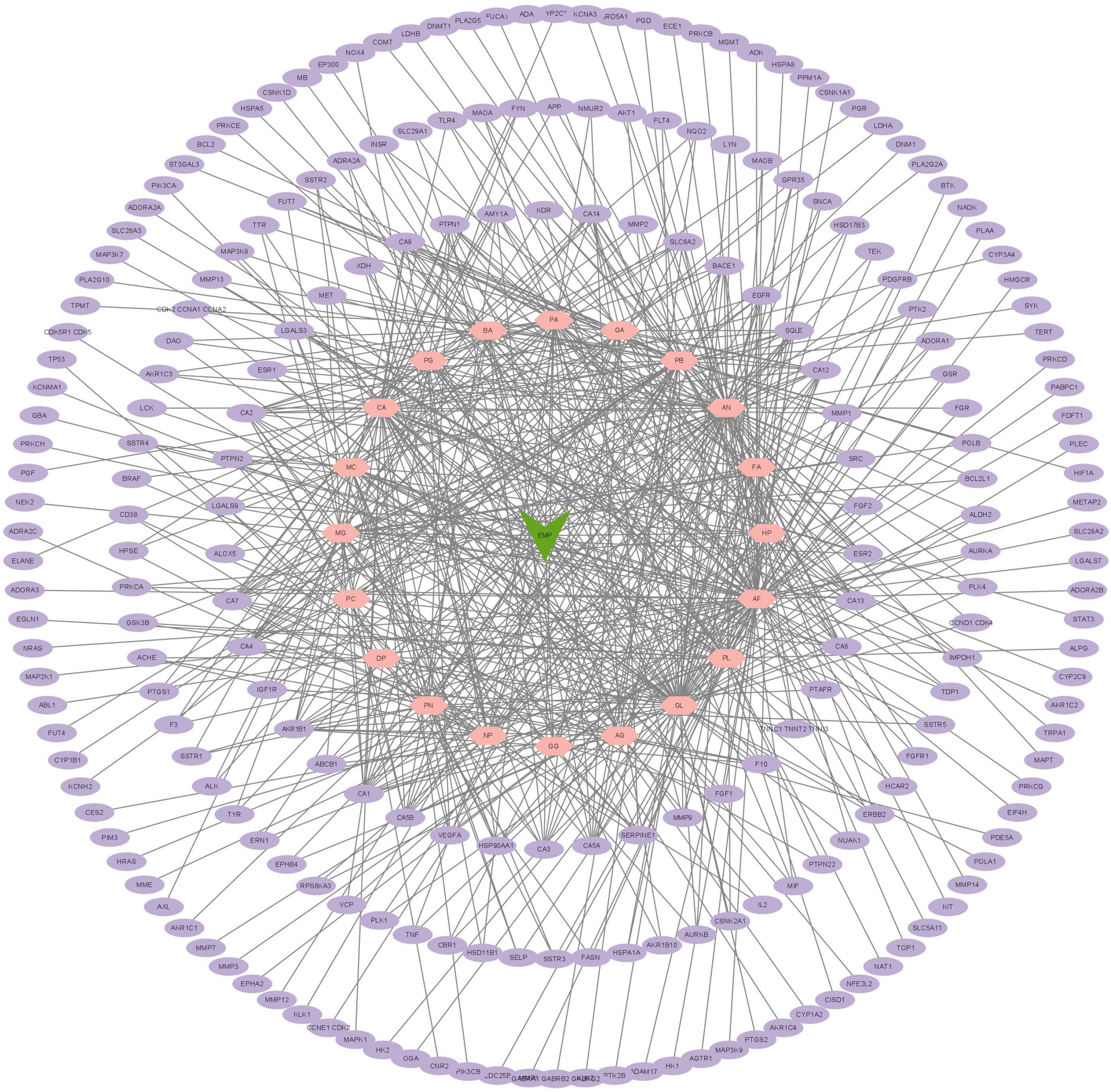

Supplementary Fig S1 Drug-active component-target network.

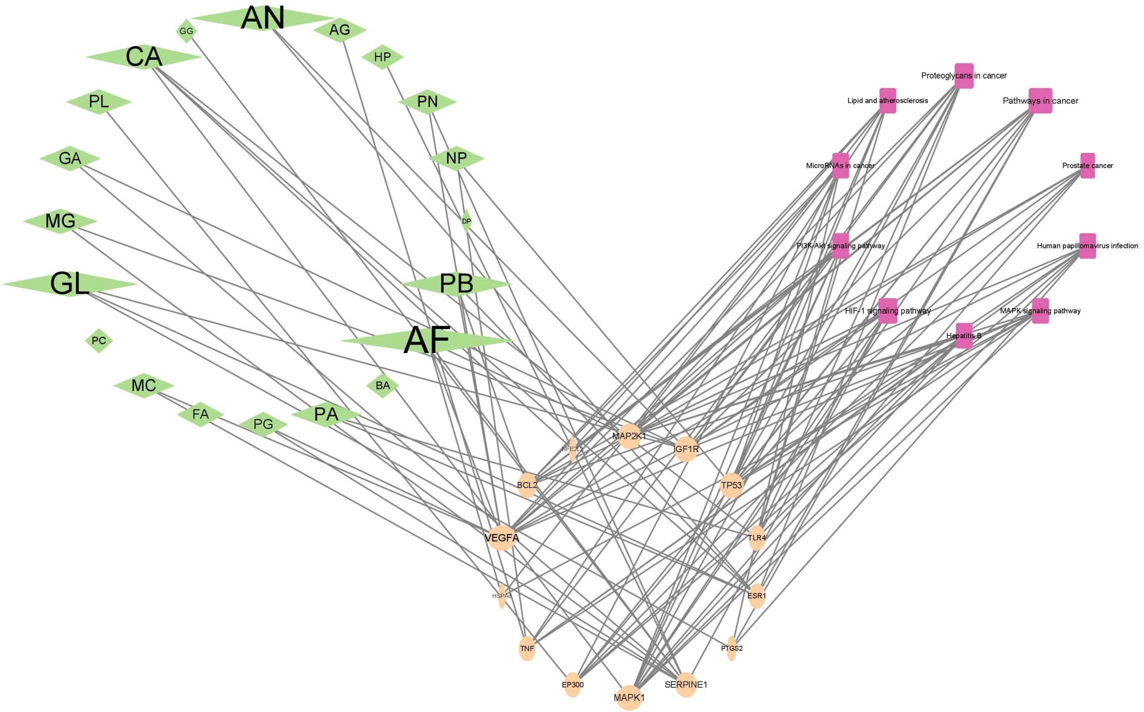

Supplementary Fig S2 The "component-target-pathway" relationship network diagram.
